# Extracellular matrix stiffness in endometrial cancer: driving progression and modulating treatment sensitivity via the ROCK1/YAP1 axis

**DOI:** 10.1038/s41419-025-07697-8

**Published:** 2025-05-14

**Authors:** Rui Sun, Ying Zhao, Yao Liu, Mengyao Zhang, Ziyi Qiu, Xiaohong Ma, Lina Wei, Wei Lu, Zhiming Liu, Jie Jiang

**Affiliations:** 1 Department of Obstetrics and Gynecology, Qilu Hospital of Shandong University, Jinan, China; 2 Gynecologic Oncology Key Laboratory of Shandong Province, Qilu Hospital of Shandong University, Jinan, China; 3 Department of Obstetrics and Gynecology, The Affiliated Yantai Yuhuangding Hospital of Qingdao University, Yantai, China

**Keywords:** Endometrial cancer, Tumour biomarkers

## Abstract

Endometrial cancer (EC) is among the most prevalent gynecological malignancies, with advanced or recurrent cases posing significant treatment challenges due to limited responses to conventional therapies. Growing evidence highlights the critical role of extracellular matrix (ECM) stiffness in driving tumor progression by shaping the tumor microenvironment. In this study, we demonstrate that ECM stiffness is significantly higher in EC tissues compared to normal endometrium, correlating with elevated expression of ROCK1, a mechanosensitive kinase. Using atomic force microscopy (AFM), we quantified ECM stiffness, while polyacrylamide gels with varying stiffness were employed to mimic ECM conditions in vitro. Bioinformatics analyses, immunofluorescence, Western blotting, and co-immunoprecipitation experiments revealed that ROCK1 modulates the phosphorylation of YAP1, promoting its nuclear localization and transcriptional activity, thereby driving aggressive tumor behaviors, including enhanced proliferation, migration, invasion, and reduced apoptosis. Pharmacological inhibition of ROCK1 with Y-27632 mitigated these effects, suppressing tumor growth, restoring apoptosis, and inducing cell cycle arrest. Treatment with Y-27632 improved sensitivity to chemotherapy and radiotherapy, and significantly enhanced macrophage-mediated phagocytosis, thereby boosting anti-tumor immune responses. In hormone-resistant EC cells, ROCK1 inhibition restored sensitivity to progesterone therapy. Notably, in vivo experiments in a xenograft mouse model confirmed the therapeutic potential of Y-27632, as combination therapy with progesterone showed superior tumor-suppressive effects compared to monotherapy. These findings underscore the dual role of ECM stiffness and ROCK1 in driving tumor progression and influencing treatment outcomes. By elucidating the relationship between ECM stiffness, ROCK1/YAP1 signaling, and treatment sensitivity, this study highlights the potential of targeting the ROCK1/YAP1 axis as a therapeutic strategy. ROCK1 serves as both a biomarker for prognosis and a target for improving personalized treatment approaches, offering new avenues to enhance clinical outcomes for EC patients.

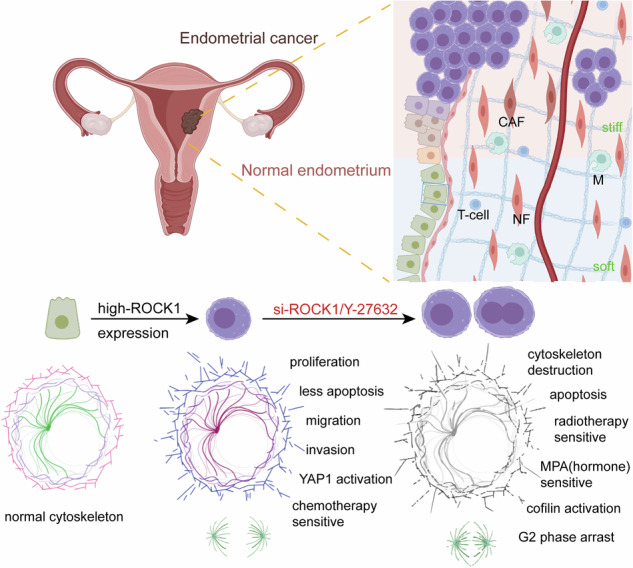

## Introduction

Endometrial cancer (EC) is the sixth most commonly diagnosed cancer in women, with 417,000 new cases and 97,000 deaths in 2020 [1]. Meanwhile, it is the most prevalent gynecological cancer in high-income countries in 2021, and its incidence is increasing globally [2]. EC patients tend to show a good prognosis due to early diagnosis. Standard-of-care first-line systemic therapy for patients with EC is conclusive and has a favorable outcome. But the prognosis of metastatic and recurrent EC is generally miserable, with a 5-year overall survival rate of 15%–18% [3]. Additionally, there hasn’t been much progress in the quest for new, effective treatments [4]. Novel treatments, while examining pertinent molecular biomarkers and key prognostic factors, are extremely important.

Extracellular matrix (ECM) is a universal scaffold for maintaining tissue and organ homeostasis. It is well acknowledged that cell behavior, fate, and function are significantly influenced by the mechanical characteristics of the extracellular microenvironment [5]. Increased ECM stiffness is strongly connected with pathological states and is regarded as a crucial component preceding tumorigenesis [6–8]. Increased ECM deposition and crosslinking is one of the main reasons for matrix stiffening. Recent technological advancements have made it possible to adjust the stiffness of biocompatible materials like polymers or gels by carefully controlling the degree of crosslinking. Polyacrylamide hydrogels can now have elastic moduli that are within the physiological range of the tissues, for example [9]. Made it possible to explore the processes of cells’ response to mechanics by precisely replicating the stiffness range of healthy and pathological tissues. Recent studies have shown that malignant tumors have been demonstrated to be much stiffer than benign tumors in a variety of cancer types, including breast [10, 11], liver [12], pancreatic [13], and prostate [14]. Several characteristics of cancer, including angiogenesis [15], metabolism [16], proliferation [17], invasion [18], migration [19, 20], metastasis [21], and immune evasion [22], depend on the stiffness of the ECM. The stiffness of the ECM has a major impact on the ability of cancerous cells to invade the basement membrane [23]. Some signaling molecules, such as integrins, focal adhesion kinase (FAK), Piezo, yes-associated protein (YAP), and Rho-associated protein kinase 1 (ROCK1), have been found to function in various types of cells in responding to ECM stiffness [24–27].

ROCK1 belongs to the AGC kinase family of Ser/Thr kinases and modulates cellular contractility by regulating myosin phosphorylation [28–32]. It has been established that ROCK1 plays a role in cellular motility and promotes cancer cell migration, invasion, and metastasis in various cancers [33]. Additionally, ROCK1 acts as a mediator of the reciprocal interactions between tumor cells and their microenvironment. Elevated expression of ROCK1 is observed in response to ECM stiffening, contributing to tissue rigidity by inducing the synthesis of collagen, fibronectin, and so on, thereby intensifying the feedback loop of ECM stiffening [34, 35]. Notably, the expression of ROCK1 is correlated with the therapeutic sensitivity of numerous diseases. For instance, reduced ROCK1 expression enhances the radiation sensitivity of cervical cancer, while elevated ROCK1 expression strengthens therapeutic resistance in colitis. Activation of the ROCK1 signaling pathway also impacts chemotherapy sensitivity of gastric, liver, and lung cancer. However, the role of ROCK1 in EC, which is intimately connected to ECM stiffness, has rarely been studied.

To elucidate the exact roles and underlying mechanisms of elevated ECM stiffness and ROCK1 expression in ECs, we conducted the following experiments. Firstly, we compared the stiffness of malignant and healthy endometrium, and conducted in vitro cellular experiments with PA gels of different stiffness. In the subsequent investigation, we identified ROCK1, a gene associated with ECM stiffness, and elucidated the underlying mechanisms by which high ROCK1 expression promotes the progression of EC. Furthermore, we also observed that patients with different levels of ROCK1 expression may exhibit different sensitivities to radiotherapy or chemotherapy. These findings shed light on a novel therapeutic avenue for EC by targeting ROCK1.

## Materials and methods

### Antibodies and reagents

Antibodies used in the present study are as follows. β-Actin (#4970), PARP1 (#9542), CyclinB1 (#12231), CyclinD1 (#2978), CDK4(#12790), cofilin1 (#5175), anti-rabbit IgG (#7074), anti-mouse IgG (#7076), Epithelial-Mesenchymal Transition (EMT) Antibody Sampler Kit (#9782) were purchased from Cell Signaling Technologies. Antibodies for CD86 (A21198), p-YAP1 (AP0489), were from ABclonal. Ribbit anti-ROCK1 antibody (ab134181), ribbit anti-ROCK2 antibody (ab125025), ribbit anti-Ki-67 antibody (ab92742), ribbit anti-CD163 antibody (ab182422), and ribbit anti-YAP1 antibody (ab76252) were purchased from Abcam. Cleaved-ROCK1 (sc-71965), phospho-cofilin1 (sc-271921) were purchased from Santa Cruz.

Y-27632 (HY-10071) was purchased from MCE. Medroxyprogesterone acetate (MPA) was obtained from Abcam. Movat-Russell modified pentachrome stain kit (G3701) from Solarbio. Actin-Tracker Red-594(C2205S) from Beyotime.

### Clinical specimens

Ninety endometrioid adenocarcinoma samples and 26 normal endometrium samples from who had hysterectomy due to other benign diseases were collected at the Department of Gynecology of the Qilu Hospital Affiliated to Shandong University. None of the patients with EC received preoperative treatment. All experiments in this study were approved by the Ethics Committee of the Qilu Hospital of Shandong University.

### TCGA data acquisition and bioinformatics

The EC gene expression data, clinical information, and mutation status were obtained from The Cancer Genome Atlas (TCGA) database. Molecular subtypes information was downloaded from the Uterine Corpus Endometrial Carcinoma (TCGA, PanCancer Atlas) dataset of cBioPortal. Standardized mRNA expression profiles of 31 solid tumors were downloaded from the Xena UCSC database (https://xena.ucsc.edu/). The cellular response to mechanical stimulus-associated gene set was downloaded from the MSigDB database (http://www.gsea-msigdb.org/gsea/msigdb/). The bioinformatics algorithm based on gene set variation analysis was quantified in 31 cancer types to evaluate the enrichment score of each sample at the pathway level, thereby characterizing the pathway activity level in each sample. Subsequently, we conducted Spearman correlation analysis between ROCK1 expression and the activity of the mechanical stimulus-associated gene pathway in each cancer type. The correlation coefficient and the significance (*P* value) of the correlation were projected onto a two-dimensional plane.

Half-maximal inhibitory concentration (IC50) is widely recognized as a crucial metric for assessing drug potency or sample treatment response. Lower IC50 values indicate enhanced drug potency, greater antitumor capacity, and heightened sensitivity to the drug’s effects. Leveraging the Genomics of Drug Sensitivity in Cancer database (https://www.cancerrxgene.org/), we employed the R package “pRRophetic” to predict potential response to chemotherapeutic agents. Notably, the studied drugs encompassed cisplatin, gemcitabine, doxorubicin, paclitaxel, etoposide, and vinorelbine. According to Eschrich’s study, a rank-based radiosensitivity index (RSI) derived from 10 genes (AR, c-JUN [JUN], STAT1, PKC [PRKCB], Rel A [RELA], cABL [ABL1], SUMO1, CDK1, HDAC1, and IRF1) was generated to predict the survival fraction at 2 Gy (SF2) across 48 cancer cell lines [36]. The prognostic value of RSI has been validated using several independent datasets, such as breast cancer, lung cancer, pancreatic cancer, and glioblastoma [37–40].

ESTIMATE analysis generates three types of scores: immune score (positively reflecting the abundance of immune cells), stromal score (positively reflecting the abundance of stromal cells), and ESTIMATE score (positively reflecting nontumor composites). We divided EC patients into two groups according to the expression of ROCK1 and ranked the genes according to the difference in expression between the two groups. Using the C2 gene set from the MSigDB database as a reference, we performed GSEA enrichment analysis with the “clusterProfiler” package and visualized the results using the “ggplot2” package. Immunophenoscore (IPS) was developed to predict the potential response of patients to immunotherapy. The IPS scores of patients were downloaded from the TCIA database (https://tcia.at/) and subsequently compared according to ROCK1 expression levels.

### Immunohistochemistry staining and Russell-Movat pentachrome staining

All clinical specimens were fixed in formalin and embedded in paraffin, cut into 4 μm sections. In brief, paraffin was removed from the tissue using xylol and dehydrated in a graded series of ethyl alcohol concentrations.

For IHC staining, antigens were retrieved by boiling with sodium citrate solution (PH 6) for 20 min in microwave. After washing in 0.05% Tween 80 in phosphate-buffered saline (PBS), the slides were incubated in methanol for 15 min and blocked with PBS containing 10% horse serum and 3% bovine serum albumin for 30 min. The sections were incubated with the appropriate antibody and transferred to the refrigerator for overnight incubation. After washing, the slides were incubated with the appropriate conjugated secondary antibody for 30 min. The slides were analyzed using a microscopic imaging analysis system (IX71, Olympus Ltd., Japan). The immunohistochemical staining was scored for both positive cells’ proportion (0 score: 0%, 1 score: < 25%, 2 score: 25–50%, 3 score: 51–75%, and 4 score: ≥75%) and staining intensity (0 score: negative, 1 score: weak, 2 score: moderate, and 3 score: strong), which ultimately resulted in designations of complete loss of expression, or weak, moderate, or strong expression, respectively.

For Russell-Movat pentachrome staining, sections were stained according to the manufacturer’s instructions.

### Tissue preparation and AFM measurements

Fresh tissue was collected, embedded with Tissue Tek Optimum Cutting Temperature Compound gel, and stored at −80 °C. Twenty-micrometer-thick frozen sections were made with a freezing microtome, and after thawing, washed three times with PBS. AFM measurements were performed in a PBS liquid environment at room temperature. Using PFQNM_LC probe. Set the parameters as follows: choose peak force QNM mode, set the probe’s elastic constant to 0.01 N/m, scanning a matrix of 20 × 20 μm, scanning amplitude at 200–400 nm, peak force frequency at 1 kHz, probe indentation force at 50–400 pN, at line speed of 0.5 Hz, a matrix image with a resolution greater than or equal to 128 × 128 bits was finally obtained. Tissue topography and elastic modulus can be obtained simultaneously in this mode.

### Cell culture

Human EC cell lines Ishikawa, HEC-1A, RL-95, AN3CA, and THP-1 were obtained from Zhong Qiao Xin Zhou Biotechnology Co., Ltd. (Shanghai, China), and KLE cells were purchased from Procell Life Science & Technology Co., Ltd. (Wuhan, China). All the above cell lines were authenticated using Short Tandem Repeat analysis. MPA was used to establish an MPA-resistant Ishikawa cell line, which we referred to as Ishikawa-MR (or “Ish-MR”). Ishikawa cells were maintained in Dulbecco’s Modified Eagle Medium (DMEM) (BI, Israel). THP-1 and Ish-MR cells were cultured in RPMI1640 medium (Gibco, NY, USA). HEC-1A cells were cultured in McCoy’s 5A medium (Gibco, NY, USA) with 1% sodium pyruvate solution. RL-95 cells were cultured in DMEM-F12 (Gibco, NY, USA) with 1% sodium pyruvate solution and 5 μg/ml insulin. AN3CA cells were cultured in MEM (Gibco, NY, USA) with 1% sodium pyruvate solution and 1% non-essential amino acid solution. KLE cells were cultured in DMEM-F12 (Gibco, NY, USA). All the media contained 10% fetal bovine serum (v/v) and 1% penicillin-streptomycin (v/v), and all the cells were cultured in an incubator containing 5% CO_2_ at 37 °C.

### Polyacrylamide gel preparation and cell culture

Polyacrylamide gels (PA) were prepared using a formulation consisting of 40% acrylamide, 2% bis-acrylamide, ammonium persulfate (APS), and tetramethylethylenediamine (TEMED). After being mixed with different ratios of deionized water, different stiffness gels were generated according to a protocol developed by Justin R. Tse [9]. Table 1 displays the stiffness and substrate details. Following formulation, the PA gel was immersed in sterilized PBS (washed more than three times) and absorbed for more than 24 h. Subsequently, 200 μl of Sulfo-SANPAH-cross-linker (abcam, ab145610) was applied to the gels’ surface, and UV light was utilized to activate the cross-linker for 20 min. Next, type I rat tail collagen was introduced onto the gels after eliminating excess liquid. The system was then incubated at 4 °C overnight to establish collagen linkage onto the gels. Washed gels with PBS three times and seeded cells on them for incubation.

**Table 1:** Composition of polyacrylamide gels with different stiffness.

Estimated G	3 kPa (μl)	30 kPa (μl)
40% Acrylamide	187.5	300
2% bis-acrylamide	54	120.5
APS	100	100
TEMED	1.5	1.5
dH_2_O	657	478
Total	1000	1000

The table shows the composition for preparing 1 ml of polyacrylamide gels with different elastic moduli (G).

*APS* ammonium persulfate, *TEMED* tetramethylethylenediamine, *dH*_2_*O* distilled water.

### RNA interference and lentivirus transfection

Lipofectamine 2000 was used for small interfering RNA (siRNA) (GenePharma Shanghai, China) transfection according to the manufacturer’s manual. The target sequences are si-ROCK1 “sense 5′-CCA GUU GUA CCC GAU UUA ATT-3′, antisense 5′-UUA AAU CGG GUA CAA CUG GTT-3′”. Si-NC “sense 5′-UUC UCC GAA CGU GUC ACG UTT-3′, antisense 5′-ACG UGA CAC GUU CGG AGA ATT-3′”. Lentivirus carrying ROCK1 or YAP1 and control lentivirus purchased from GeneChem (Shanghai, China) were transfected into Ishikawa and Ish-MR cells according to the manufacturer’s manual.

### Western blotting and immunoprecipitation

Harvest cells at the logarithmic growth stage, washed three times with PBS (pre-cooled at 4 °C) and lysed [radioimmunoprecipitation assay lysis buffer (Beyotime Institute of Biotechnology, Haimen, China), phenylmethanesulfonyl fluoride, and phosphatase inhibitor at a ratio of 100:1:1] for 30 min on ice. The lysates were collected and centrifuged at 12,000 × *g* for 15 min at 4 °C. Then, a bicinchoninic acid protein assay was used to detect the protein concentration. The Western blot assay was carried out as previously described [41].

For co-immunoprecipitation, cell lysates of were incubated with IgG and specific antibodies (2 μg) for 14 h at 4 °C with continuous inverted rotation. Subsequently, 20 μl of protein G agarose beads were added into the lysates, maintaining a constant rotation for an additional 4 h. After centrifugation, the beads were retained, and after three rounds of RIPA washing, 25 μl of 1x protein loading buffer was added. Beads were denaturized in a metal bath at 100 °C for 5 min, and the supernatant protein was separated through SDS-PAGE and visualized using the western blot methodology.

### Total RNA isolation and qTR-PCR

Cells’ Total RNA was extracted using TRIzol reagent (Invitrogen, Carlsbad, CA, USA) according to the manufacturer’s recommendation. The concentration and purity of the extracted RNA were detected using a spectrophotometer (Thermo Fisher Scientific Inc., MA, USA). Then the RNA was transcribed into cDNA. qRT-PCR was performed to quantify RNA expression using the SYBR Green Master Mix (Takara, Japan) on a StepOne™ PCR amplifier (Applied Biosystems, USA). Primers used included (listed 5′ to 3′): β-Actin-F’: GAA GAG CTA CGA GCT GCC TGA; β-Actin-R’: CAG ACA GCA CTG TGT TGG CG; ROCK1-F’: GGA AGT GAG GTT AGG GCG AA, ROCK1-R’: ACA GTG TCT CGG AGC GTT TC. YAP1-F’: TCC CTC GAA CCC CAG ATG AC, YAP1-R’: TGT CCC AGG AAT GGC TTC AA.

### Colony formation assay

Transfected cells were seeded in 6-well plates at a density of 300 cells per well. They were then incubated in an incubator at 37 °C for 14 days, fixed with 4% paraformaldehyde for 20 min, and stained with crystal violet (Beyotime, Beijing, China) for 1 h at room temperature. The colonies were quantified by ImageJ software.

### Cell proliferation assay (CCK-8 assay and MTT assay)

Transfected cells were seeded into 96-well plates at a concentration of 2000 cells per well, with 100 μl of complete medium for cell proliferation assay and 100 μl of medium containing 1% fetal bovine serum for drug susceptibility testing. For the CCK-8 cell proliferation assay, cells were harvested at 0 h, 24 h, 48 h, 72 h, and 96 h, respectively, after being cultivated at 37 °C. And for the drug susceptibility test, cells were harvested after 48 h of drug incubation. The detailed procedure was as described previously [42].

### Cell immunofluorescence and F-actin staining

The cells in the 96-well plate underwent diverse treatments before being fixed with 4% paraformaldehyde for 30 min. Subsequently, cells were incubated with 3% glycine for 10 min, washed three times with 1x PBS, then blocked with 3% BSA for 30 min before moving on to the next steps.

For cell immunofluorescence, the cells were exposed to the target antibody at a concentration of 1:200 for an overnight incubation at 4 °C. The cells were treated with a particular fluorescent secondary antibody at 37 °C for 1 h on the second day, following three PBS washes. After the nuclei were stained with 4,6-diamidino-2-phenylindole (DAPI) staining solution for 10 min, cells were imaged under a fluorescence microscope.

For F-actin staining, Actin-Tracker Red-594 was purchased from Beyotime Biotechnology. After 1 h of F-actin tracker working solution (1:40 diluted) staining, the cells were washed three times with PBS, stained for 10 min with DAPI, and then analyzed using microscopic imaging analysis system (IX71, Olympus Ltd., Japan) and the mean fluorescence intensity for 4–6 zones was calculated using ImageJ/ NIH software.

### Transwell assay

To conduct transwell migration and invasion assay, approximately 15-20 × 10^4^ cells precultured in 200 μl serum-free media were seeded into the upper compartment of Transwell chambers equipped with 8-µm pores (Corning Life Sciences). For invasion experiments, a mixture of 70 μl of 1:9 diluted Matrigel (BD Biosciences) was added to the upper chamber to solidify overnight at 37 °C before plating cells. Medium containing 10% FBS was added to the lower chambers. The entire setup was then placed within a 5% CO_2_ incubator at 37 °C for 20 h and 36 h, respectively, for the migration and invasion assay. A cotton swab was used to gently remove the cells from the upper chamber. The cells left on the transwell membrane were then fixed in methanol and stained with hematoxylin. And then captured with a microscopic imaging analysis system (IX71, Olympus Ltd., Japan)

### Flow cytometry (cell apoptosis assay and Cell cycle assay)

At the designated time points, cells from different treatment groups were harvested and washed twice with PBS. Then, in accordance with the manufacturer’s manual, a cell cycle distribution assay was performed using propidium iodide (PI, BD Biosciences) staining solution. Additionally, cell apoptosis assessment was performed using a fluorescein isothiocyanate (FITC) Annexin V Apoptosis kit (BD Bioscience). Finally, CytoFLEX Flow Cytometer (Beckman Coulter, USA) analysis was performed.

### Cell irradiation treatment

Cells in the logarithmic growth phase were seeded into 6-well plates and exposed to a 6 MV X-ray irradiator (Clinac-23-EX, Varian, USA) at room temperature. We initially used a total dose of 0, 3, 6, 9 Gy to choose an appropriate condition. Finally, 6 Gy was selected as the final radiotherapy dose of the experiment.

### THP-1 cell differentiation, polarization, and phagocytosis assay

THP-1 cells were seeded into 6-well plates and exposed to 100 nM of phorbol-12-myristate-13-acetate (PMA, Beyotime, S1819) for 48 h for adherence, followed with 100 ng/ml LPS and 20 ng/ml IFN-γ for another 48 h to generate M1-polarized THP-1 macrophages.

For phagocytosis assay, M1 macrophages were marked with Cell-Tracker CM-Dil (Invitrogen) for 10 min at 37 °C, and co-cultured with CFSE-dyed (MCE, #79265) tumor cells in serum-free 1640 medium in the presence or absence of Y-27632 (30 μM). After incubation at 37 °C for 3 h, cells were analyzed by CytoFLEX Flow Cytometer (Beckman Coulter, USA).

### Tumor xenograft model

The female BALB/c nude mice (6–8 weeks) used in this study were treated in compliance with ethical approvals of Shandong University and maintained in Specific Pathogen-Free (SPF) conditions. The mice were procured from Beijing Vital River Laboratory Animal Technology. 1 × 10^7^ tumor cells were injected subcutaneously, and after tumor formation, the mice were separated into four groups and intraperitoneally injected every other day. The four groups were the normal saline group, the MPA (100 mg/kg/body weight) group, the Y-27632 (10 mg/kg/body weight) group, and the MPA plus Y-27632 combination group. Tumor volumes were calculated using the formula *V* = [(length × width^2^)/2]. Subsequent to the sacrifice of the mice, tumor weight was measured.

### Statistical analysis

Statistical analysis was carried out using GraphPad Prism 8 and R software. Student’s *t*-test, Chi-square test, and Kaplan–Meier survival analysis were performed. All data were expressed as mean ± standard deviation (SD), and three independent replications were performed. *P* value < 0.05 was considered to be statistically significant.

## Results

### The ECM of EC patients is stiffer than that of normal endometrium

**Fig. 1:**
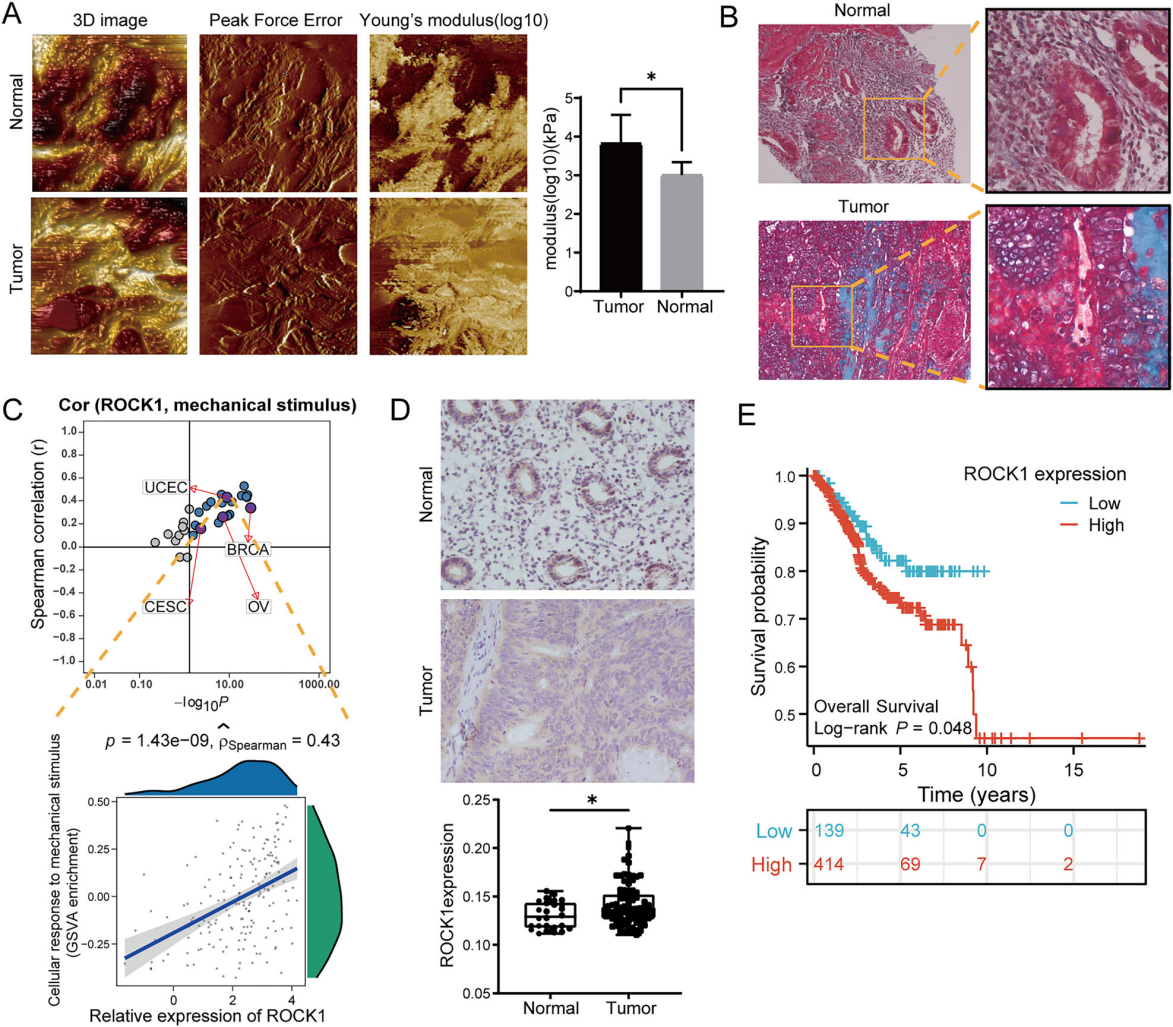
Endometrial cancer, ECM stiffness, and ROCK1 expression analysis. **A** Stiffness of the ECM in normal endometrium and endometrial cancer detected by atomic force microscopy (AFM) QNM mode. 3D images, peak force error maps, Young’s modulus maps, and statistical graphs were generated for analysis. **B** Russell-Movat pentachrome staining of normal endometrium and endometrial cancer tissue. **C** Pan-cancer analysis of the correlation between ROCK1 expression and mechanical stimuli-related gene sets (colored points present for positive correlation, with pan-gynecological cancer in purple). The graph below illustrates the correlation between ROCK1 expression and gene sets associated with mechanical stimuli in each patient with endometrial cancer. **D** IHC staining of ROCK1 in normal endometrium and endometrial cancer tissue. Statistics graph of IHC. **E** Kaplan–Meier curves for overall survival in EC patients with different ROCK1 expression. *P < 0.05, ***P < 0.001.

In assessing the stiffness of endometrium ECM, we employed AFM to detect the Young’s modulus of ECM in both EC and noncancerous patients. As shown in Fig. 1A, EC patients were found to have stiffer ECM than normal endometrium. The Movat-Russell modified pentachrome stain method was used to distinguish the components of ECM between normal endometrial and EC samples. And it shows the cellulose was significantly increased in patients with EC (Fig. 1B). Next, to delve deeper into the underlying mechanism driving heightened ECM stiffness in EC patients, we conducted analyses using publicly available datasets. Remarkably, we found that the expression of ROCK1 was positively correlated with mechanical stimulation across multiple cancers, and among the pan-gynecological cancers (comprising breast cancer, ovarian cancer, cervical cancer and EC), EC had the highest correlation scores (Fig. 1C). Moreover, we found that ROCK1 expression was higher in EC patients compared to individuals with normal tissues through IHC staining (Fig. 1D). We then performed survival analysis on EC samples from the TCGA database. Intriguingly, individuals with high ROCK1 expression displayed a poorer prognosis (Fig. 1E). We employed the Estimate algorithm to assess immune, stromal, and tumor purity scores of EC patients. Interestingly, we observed that patients with high ROCK1 expression had elevated tumor purity, while those with low expression exhibited higher immune scores (Fig. S1A–C). GSEA enrichment analysis revealed that patients with high ROCK1 expression were predominantly enriched in the cell cycle pathway, suggesting enhanced cell proliferation capability in these patients (Fig. S1D). Based on the above findings, we further investigated the impact of ROCK1 expression on EC cells.

### Increased ECM stiffness promotes EC progression, upregulates ROCK1, and exacerbates the malignant phenotype of EC cells

**Fig. 2:**
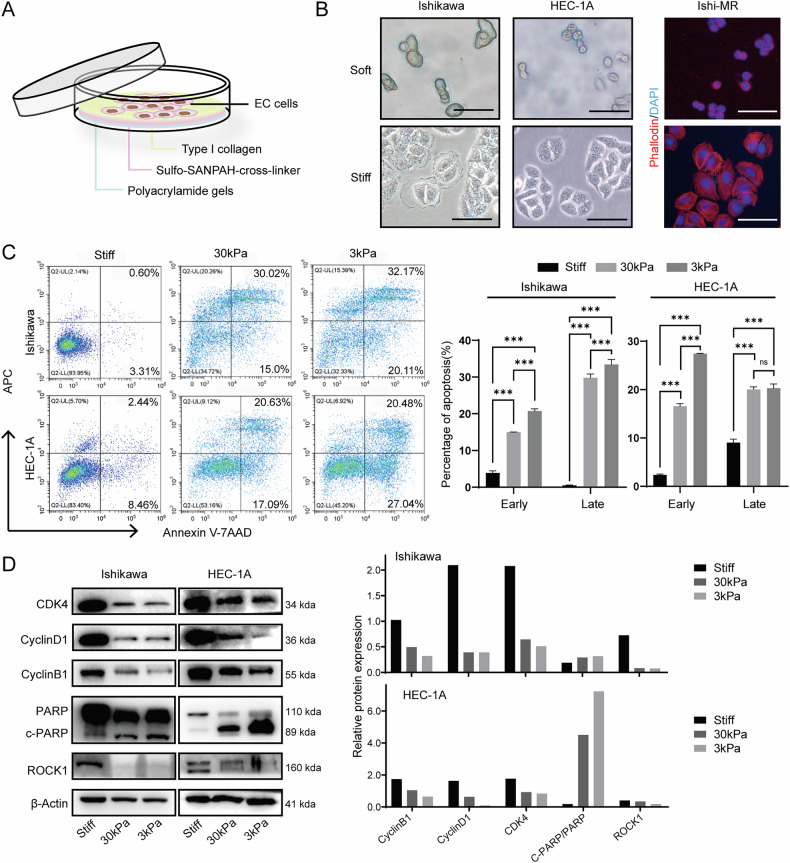
Stiff substrate induced malignant cellular phenotypes and elevated ROCK1 expression. **A** The schematic of cell culture on a polyacrylamide Gel. **B** Cell morphology and cytoskeleton staining of endometrial cancer cells on soft and stiff substrates. Scale: 50 μm. **C** Cell apoptosis ratio and statistical analysis of cells cultured on different stiff substrates. **D** The expression of ROCK1, PARP, cleaved-PARP, cyclinB1, cyclinD1, and CDK4 in Ishikawa and HEC-1A cells cultured with different stiffness substrates. ***P < 0.001. Error bar refers to the mean and SD.

Cells were cultured on different stiffness PA gels (depicted in Fig. 2A). As shown in Fig. 2B, cells cultured on soft and stiff substrates exhibit significant differences in morphology, with cells on soft substrates showing a reduction in cell area. The F-actin staining figures on the right show the cytoskeleton of cells. It shows that the cells cultured on the soft gel exhibited diminished stretching, and the cytoskeleton appeared contracted around the nucleus. Cell apoptosis assay showed an increased proportion of apoptotic cells on the soft substrates (Fig. 2C). Next, we extracted proteins from cultured cells on different substrates. Western blot experiments revealed a robust correlation between the expression of ROCK1 and substrate stiffness, showing an increase in expression with higher substrate stiffness (Fig. 2D). However, it is important to note that when the stiffness of the EC microenvironment changed, the expression of YAP1 did not exhibit significant alterations (Fig. S2). Concurrently, we also found that the expression of cell cycle-related proteins cyclinB1, cyclinD1, and CDK4 proteins was positively correlated with substrate stiffness, while the expression of apoptosis-related protein Cleaved-PARP was negatively correlated with it (Fig. 2D).

Next, to further investigate whether increased ECM stiffness in EC patients affects cell function by regulating the expression of ROCK1, we overexpressed ROCK1 and then cultured the cells on low-stiffness hydrogels. We found that the proportion of cell apoptosis was reduced compared to the control group (Fig. S2A, B), indicating that ROCK1 overexpression played a protective role. Additionally, Western blot analysis revealed that the expression of the apoptosis-related protein cleaved-PARP was also reduced in cells cultured on soft matrices after ROCK1 overexpression (Fig. S2C).

**Fig. 3:**
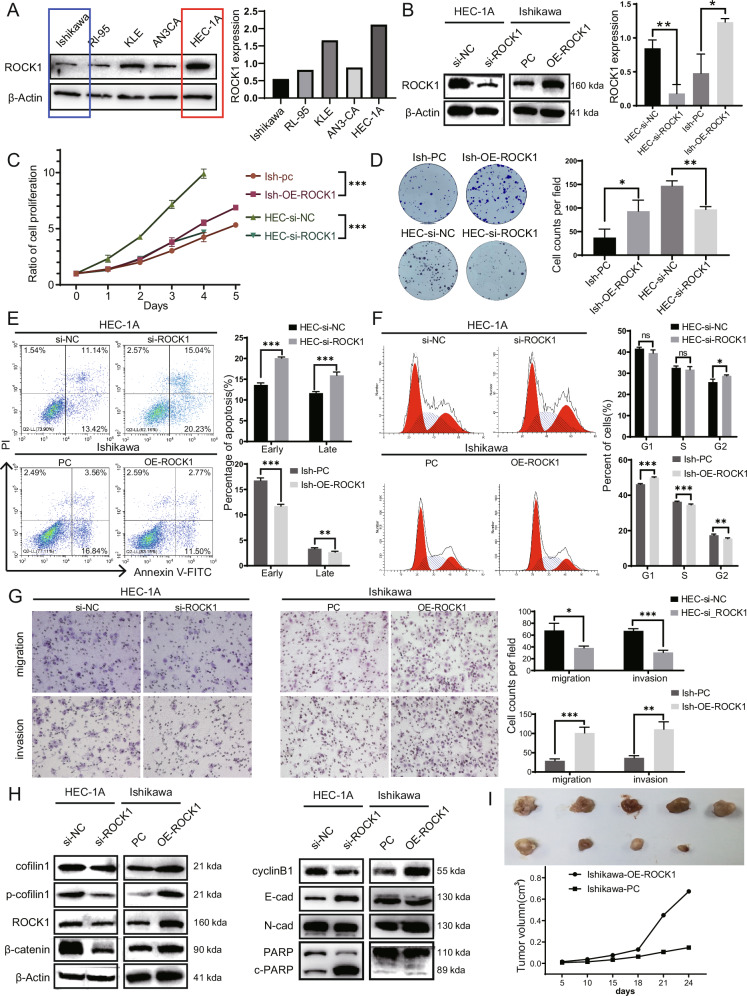
Elevated ROCK1 expression contributes to the malignant biological behavior of endometrial cancer cells. **A** Western blot analysis to detect ROCK1 expression in five endometrial cancer cell lines. **B** ROCK1 protein expression in ROCK1-interfered HEC-1A cells and ROCK1-overexpressing Ishikawa cells. (*Note: The ROCK1 band for the Ishikawa group in this panel is reused in* (**H**)*, as both panels show protein expression under the same treatment conditions (PC and OE-ROCK1)*.). **C** CCK-8 assay was used to detect the effect of ROCK1 expression on cell proliferation. **D** Colony formation ability of ROCK1-overexpressed and control Ishikawa cells. **E** Apoptosis of cells with different ROCK1 expression analyzed by flow cytometry. **F** Distribution of the cell cycle with different ROCK1 expression analyzed by flow cytometry. **G** Effect of ROCK1 expression on cell migration and invasion assessed by Transwels assay. **H** Western blot analysis was performed to analyze related protein expression. **I** Tumor growth curves of Ishikawa cells overexpressing ROCK1 and control Ishikawa cells in xenograft mice. *P < 0.05, **P < 0.01, ***P < 0.001, Error bar refers to mean and SD.

Subsequently, we evaluated the expression of ROCK1 in five EC cell lines and found that Ishikawa cells had the lowest expression and HEC-1A cells had the highest expression (Fig. 3A). Therefore, we selected the most efficient interfering RNA (siRNA) based on interference efficiency (Fig. S3A) and employed it to silence ROCK1 expression in the HEC-1A cell line. And in the Ishikawa cell line, we established a stably overexpressing of ROCK1 through lentivirus-mediated gene delivery. After verifying the efficiency of interference and overexpression of ROCK1 by western blot and RT-qPCR experiments (Fig. 3B), the subsequent experiment was carried out. The results of CCK-8 and colony formation experiments showed that interfering with ROCK1 expression in HEC-1A cells substantially attenuated their proliferation, while Ishikawa cells overexpressing ROCK1 exhibited accelerated proliferation (Fig. 3C, D). The results of flow cytometry cell apoptosis and cell cycle experiments suggested that high expression of ROCK1 hindered cell apoptosis, and inhibited the expression of ROCK1 led to cell cycle arrest in G2 phase (Fig. 3E, F). Interestingly, the high expression of ROCK1 significantly promoted cell invasion and migration capabilities. Compared with the control group, si-ROCK1 led to reduced cell invasion and migration, while OE-ROCK1 resulted in an increased number of cells passing through the chamber membrane (Fig. 3G). At the same time, Western blot analysis of proteins related to apoptosis, cell cycle and EMT revealed alignment with the cellular functional experiment, that is, the expression of cleaved-PARP and E-cadherin was negatively correlated with ROCK1, while the expression of cyclinB1, N-cadherin, and β-catenin was positively correlated with ROCK1 (Fig. 3H). Finally, we performed in vivo experiments of subcutaneous tumorigenesis in nude mice using Ishikawa-OE-ROCK1 and control cells. The results showed that overexpression of ROCK1 facilitated tumorigenesis in vivo (Fig. 3I).

### Upregulated ROCK1 promotes the progression of EC through YAP1 activation

**Fig. 4:**
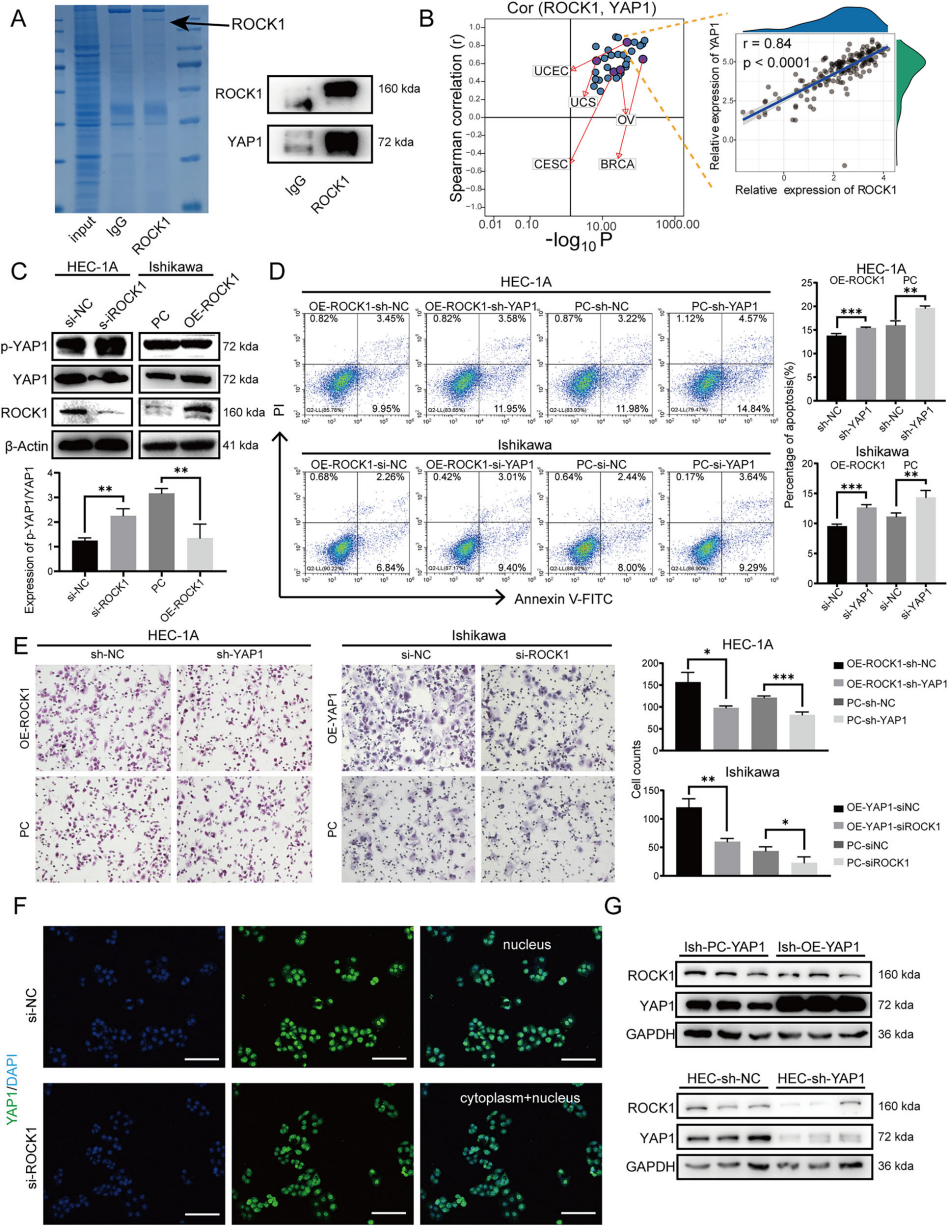
ROCK1 promotes EC progression by regulating the phosphorylation of YAP1. **A** Co-immunoprecipitation (Co-IP) experiment with ROCK1 antibody, followed by Coomassie brilliant blue staining and western blot analysis. **B** Correlation analysis of ROCK1 and YAP1 in pan-cancer, with the graph on the right showing the individual correlation between ROCK1 expression and YAP1 in each patient with endometrial cancer. **C** Western blot analysis was performed to detect the expression of YAP1 and phosphorylated YAP1(p-YAP1) in cells with different levels of ROCK1 expression. **D** Cell apoptosis experiments demonstrated the changes in apoptosis rescue by YAP1 in cells with different levels of ROCK1 expression. **E** Transwell assays were conducted to assess the changes in cell migration ability following YAP1 rescue after ROCK1 modulation. **F** Immunofluorescence analysis was performed to detect the expression of YAP1 in cells with different levels of ROCK1 expression. **G** The expression of ROCK1 was detected by western blotting in YAP1 knockdown and overexpression cells. *P < 0.05, **P < 0.01, ***P < 0.001, Error bar refers to mean and SD.

To further investigate the mechanisms underlying the effects of increased ROCK1 expression on the progression of EC, we performed a series of experiments and assessments aimed at elucidating the potential pathways involved. Initially, we embarked on a search for proteins that interact with ROCK1 through Co-IP experiments, leading to the surprising discovery of a binding partnership between YAP1 and ROCK1 (Fig. 4A). Next, in order to shed light on the role of ROCK1 and YAP1, we conducted two distinct analyses. First, we analyzed the gene expression profiles of 33 cancer types from the TCGA database and found that the expressions of ROCK1 and YAP1 were positively correlated. Specifically, this correlation was particularly strong in EC, with an *R* value of 0.84 (Fig. 4B). Then, we conducted cellular assays to deepen our understanding. Western blot experiments found that in overexpressed Ishikawa-OE-ROCK1 cells, the expression of YAP1 remained unchanged, but the phosphorylated YAP1 decreased (Fig. 4C). Subsequently, we conducted rescue experiments by knocking down YAP1 in Ish-OE-ROCK1 cells, which resulted in increased apoptosis and diminished cell invasion and migration abilities, while the cell cycle was not significantly affected (Fig. 4D, E). Parallel outcomes were achieved when overexpressing ROCK1 and silencing YAP1 in HEC cells (Fig. 4D, E).

We further confirmed through WB experiments that matrix stiffness did not affect the expression of YAP1, but phosphorylated YAP1 was negatively correlated with matrix stiffness (Fig. S2D). Immunofluorescence staining showed that when ROCK1 expression was knocked down in HEC cells, the nuclear localization of YAP1 was relatively reduced (Fig. 4F). In addition, we overexpressed YAP1 in Ishikawa cells and knocked down YAP1 in HEC-1A cells. Western blot analysis showed that ROCK1 expression remained predominantly unaltered (Figs. 4G and S4A, B). These results collectively indicate that ROCK1 expression is not regulated by YAP1, but ROCK1 dose have capability to modulate YAP1 phosphorylation. Subsequent cellular functional experiments targeting downstream YAP1 revealed that high YAP1 expression promoted cell proliferation, suppressed apoptosis, facilitated cell through G2 phase of cell cycle, and enhanced cell migration (Fig. S4). Taken together, our findings indicate that ROCK1 overexpression promotes the malignant phenotype of EC by activating YAP1, and that knocking down YAP1 can partially rescue the malignant biological behavior caused by ROCK1 overexpression.

### ROCK1 stabilizes the cytoskeleton, and Y-27632 interferes with cytoskeleton formation and inhibits EC cell migration by inactivating ROCK1

**Fig. 5:**
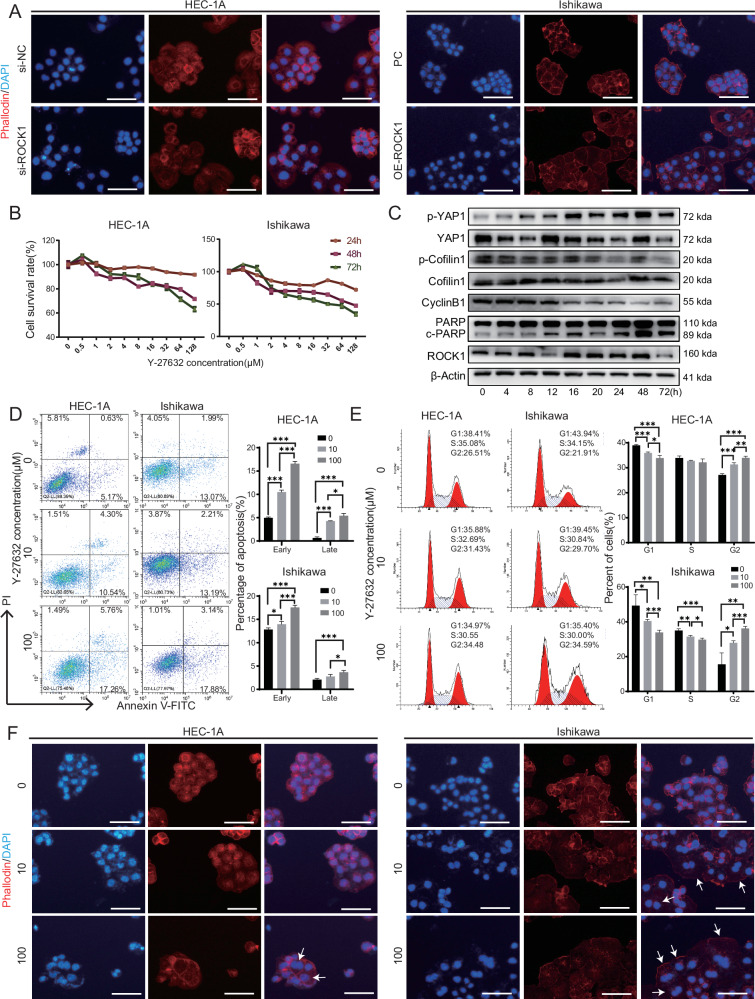
Y-27632 inactivates ROCK1 and interferes with cytoskeleton formation. **A** Immunofluorescence staining of the cell cytoskeleton with different levels of ROCK1 expression using phalloidin staining. **B** Growth inhibition curves of HEC-1A cells and Ishikawa cells treated with Y-27632 for 24, 48, and 72 h, respectively. **C** Expression of ROCK1 and related proteins after treatment with a time gradient of 10 µM Y-27632 in Ishikawa cells. **D** The impact of 0, 10, and 100 µM Y-27632 on HEC-1A and Ishikawa cell apoptosis was evaluated by flow cytometry. **E** The impact of 0, 10, and 100 µM Y-27632 on HEC-1A and Ishikawa cell cycle was evaluated by flow cytometry. **F** The impact of 0, 10, and 100 µM Y-27632 on HEC-1A and Ishikawa cell cytoskeleton was evaluated by immunofluorescence. Scale: 50 μm. *P < 0.05, **P < 0.01, ***P < 0.001, Error bar refers to mean and SD.

We performed cellular cytoskeleton staining on cells with different levels of ROCK1 expression and found that Ish-OE-ROCK1 and HEC-siNC cells had well-formed cell skeletons with longer and more orderly microfilaments (Fig. 5A). Y-27632, a selective ATP-competitive ROCK1 inhibitor, was used in CCK-8 proliferation assays. The cytotoxic effects of Y-27632 were observed to be highly time-dependent, as even at high concentrations, significant cell death was not induced after 24 h of treatment. At a dose of 10 μM, the drug had a stable effect on cell proliferation after 48 h of treatment (Fig. 5B). Employing a time-gradient approach with a 10 μM concentration of Y-27632, we found that ROCK1 expression remained unaffected. However, after 48 h of treatment, it significantly inhibited the phosphorylation of downstream factor cofilin1 [43, 44] and promoted the phosphorylation of YAP1 (Fig. 5C). Therefore, a 48-h treatment duration was selected for subsequent experiments. Apoptosis assays showed that as drug concentration increased, Y-27632 increased the rate of cell apoptosis (Fig. 5D). Cell cycle experiments showed that the G2 phase cell cycle arrest caused by Y-27632 increased with increasing drug concentration (Fig. 5E). Through cell cytoskeleton staining experiments, it became evident that the action of Y-27632 led to pronounced disruptions in the cell (Figs. 5F and S5).

Taken together, these experimental results underscore the pivotal role of ROCK1 in cell cytoskeleton formation. Additionally, Y-27632 was found to inhibit cell proliferation, promote apoptosis, induce cell cycle arrest at the G2 phase, and disrupt cell cytoskeleton formation by inhibiting ROCK1 function.

### ROCK1 is a predictor for EC patients’ adjuvant therapy sensitivity, Y-27632 enhances antitumor immunity by promoting macrophage-mediated clearance of cancer cells

**Fig. 6:**
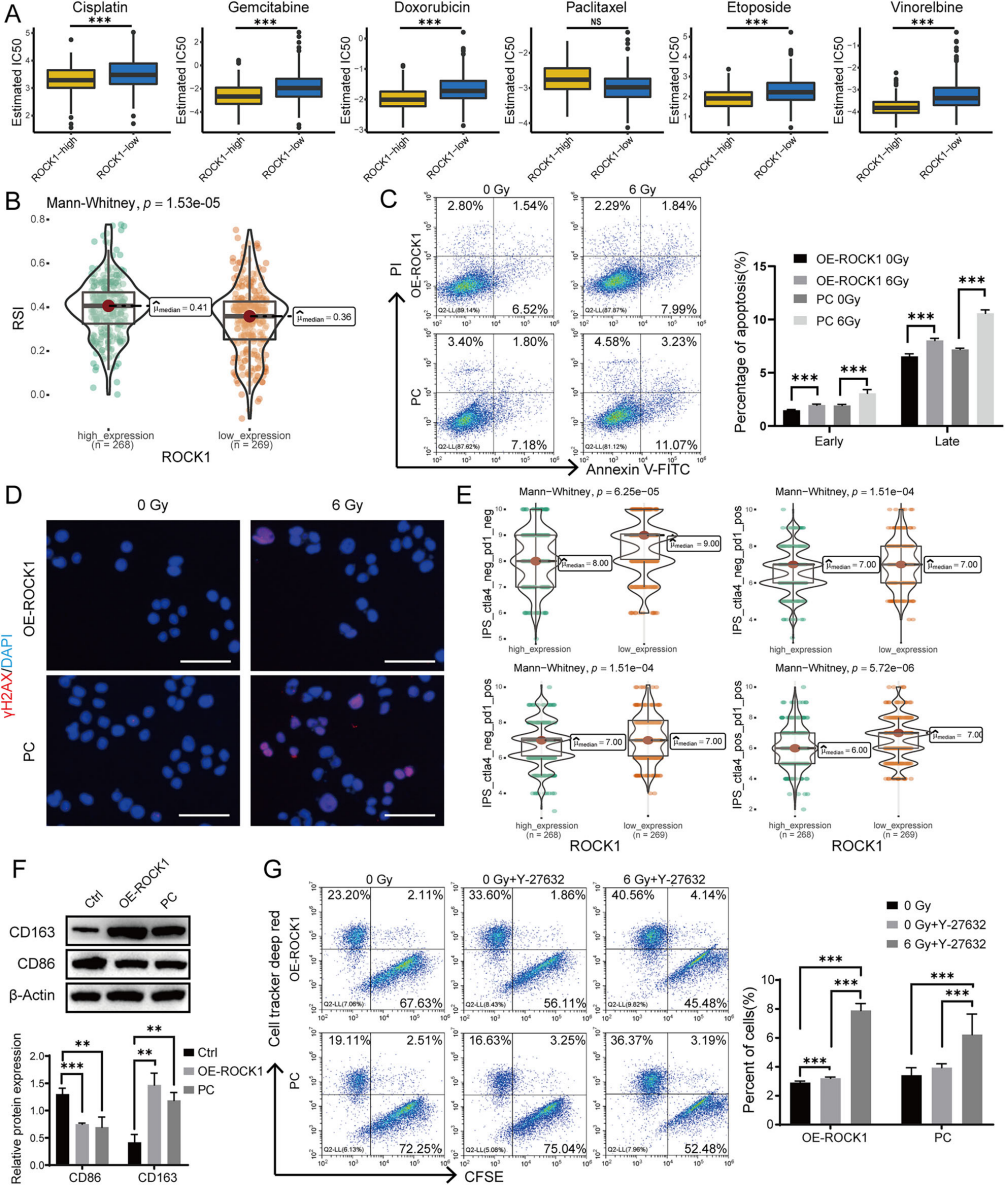
Adjuvant therapy sensitivity investigation based on ROCK1 expression. **A** Calculation of IC50 values for six chemotherapy drugs in patients with high and low ROCK1 expression to predict chemotherapy sensitivity. **B** Prediction of radiotherapy sensitivity in patients based on ROCK1 expression using the Radiotherapy Sensitivity Index (RSI). **C** Changes in apoptosis rates in Ishikawa ROCK1-overexpressing and control cells after receiving radiotherapy. **D** Expression of γH2AX in Ishikawa ROCK1-overexpressing and control cells after receiving radiotherapy. Scale: 50 μm. **E** Prediction of immunotherapy in patients with high or low ROCK1 expression using the TCIA database. **F** Expression of macrophage markers CD163 and CD86 after co-culture of THP-1 cells with conditioned medium from ROCK1-overexpressing, control, and blank control cells for 48 h. **G** CFSE-labeled tumor cells were co-cultured with cell-tracker-deep-red-labeled macrophages, Y-27632-treated for 2 h, and then analyzed by flow cytometry. Scale: 50 μm. *P < 0.05, **P < 0.01, ***P < 0.001, Error bar refers to mean and SD.

Next, we investigated the impact of ROCK1 expression levels on treatment decisions for patients. Leveraging mRNA expression data sourced from the TCGA database, we calculated chemotherapy and radiotherapy sensitivity scores. Notably, our findings unveiled that patients with high ROCK1 expression displayed diminished IC50 values for cisplatin, gemcitabine, docetaxel, etoposide, and paclitaxel, indicating a heightened responsiveness to these drugs (Fig. 6A). The radiotherapy sensitivity score calculation results showed that patients with low ROCK1 expression manifested reduced radiotherapy tolerance, indicating higher sensitivity to radiotherapy (Fig. 6B). Cell irradiation experiments unveiled a correlation between high ROCK1 expression and augmented radioresistance. This was substantiated by a decreased proportion of apoptotic cells following exposure to an equivalent 6 Gy radiation dose (Fig. 6C). Immunofluorescence staining of γH2AX revealed that cells with high ROCK1 expression exhibited less DNA damage after irradiation (Fig. 6D), further confirming the upper finding. The IPS score from the TCIA database indicated that patients with low ROCK1 expression manifested heightened immunogenicity and favorable response to immunotherapy (Fig. 6E). Furthermore, when THP-1 cells were cultured in conditional medium, overexpression of ROCK1 induced an increase in CD163 expression thereby fostering M2 polarization (Fig. 6F). Previous studies have found that Y-27632 can enhance the phagocytic activity of macrophages [45]. We used CFSE to stain tumor cells with green fluorescence and Cell-Tracker to stain THP-1 cells induced to adhere to the wall for 72 h with red fluorescence. After adding tumor cells to M0 cells for co-culture for 2 h, flow cytometry experiments showed that the phagocytic ability of cells was enhanced after treatment with Y-27632 when the cells were pre-treated with radiotherapy (Fig. 6G). The above results suggest that ROCK1 expression can serve as a predictive biomarker for patient treatment sensitivity, and that Y-27632 can enhance the phagocytic activity of macrophages.

### Elevated ROCK1 expression induced progesterone resistance in endometrial cancer cells

**Fig. 7:**
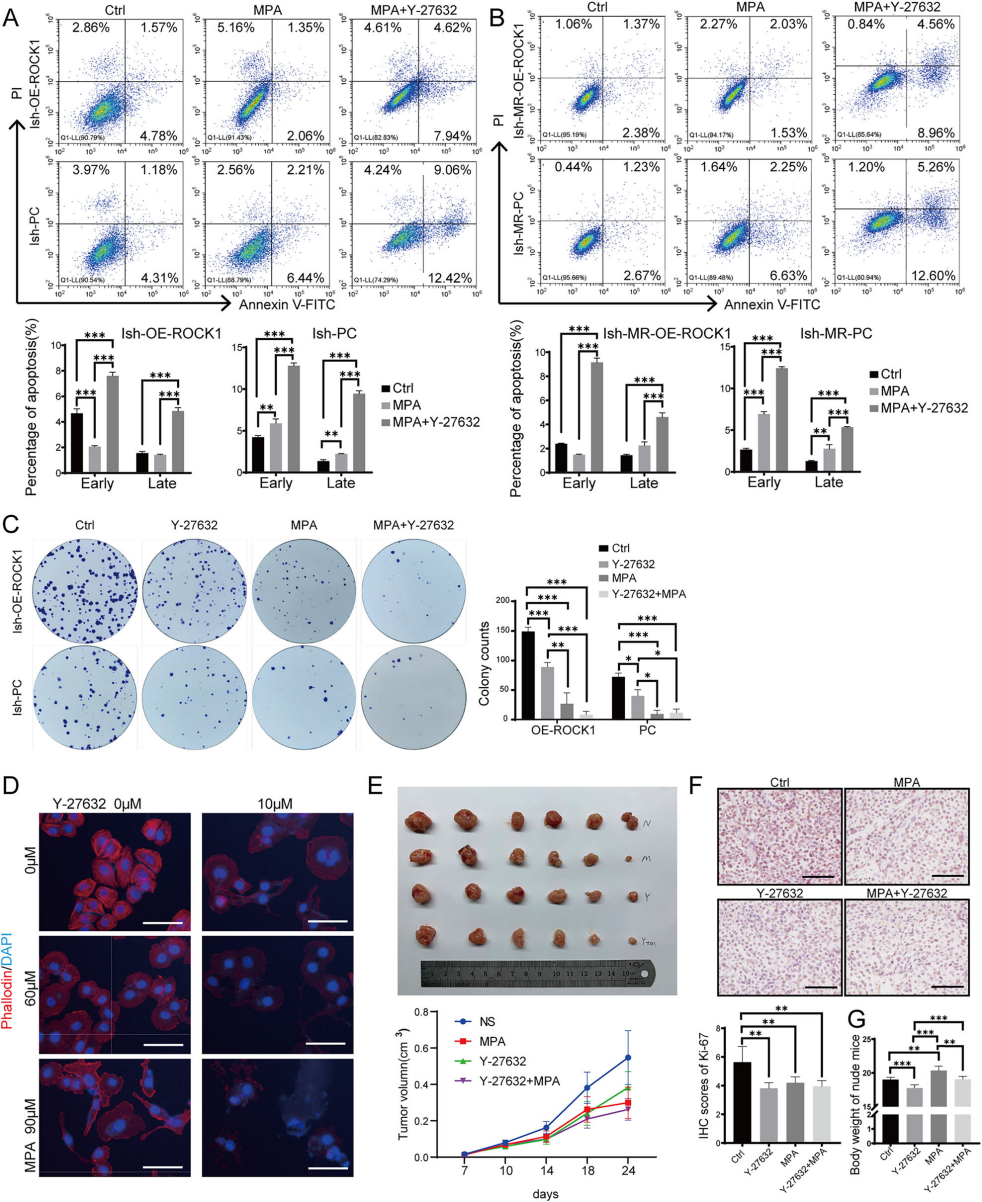
Elevated ROCK1 expression induced progesterone resistance in EC cells. **A** Cell apoptosis in ROCK1-overexpressing and control cells after treatment with MPA, Y-27632, or their combination. **B** Cell apoptosis in Ish-MR cells after treatment with MPA, Y-27632, or their combination. **C** Colony formation assay of cells treated with MPA, Y-27632, or their combination. **D** Immunofluorescence staining of the cell cytoskeleton in cells treated with MPA, Y-27632, or their combination. (*Note: The immunofluorescence image for the Y-27632 0* *μM in this panel is also presented in* Fig. 2B*(Ishi-MR Stiff group)*, *as both represent the same experimental group under identical treatment*.). **E** In vivo xenograft experiments to evaluate the effects of MPA, Y-27632, and their combination on tumor growth. **F** IHC staining of Ki-67 in mouse tumors. **G** Body weight measurements of mice prior to sacrifice. Scale: 50 μm. *P < 0.05, **P < 0.01, ***P < 0.001, Error bar refers to mean and SD.

Our previous studies have indicated a correlation between YAP1 expression and progesterone sensitivity. In light of this, we conducted progesterone sensitivity experiments using Ish-MR cells, an Ishikawa-induced progesterone-resistant cell line we established before. We measured the IC50 values of Ishikawa, HEC-1A, AN3CA, and RL-95 cell lines after MPA treatment. Among these, HEC-1A exhibited the highest IC50 value (Fig. S6A, C). Compared to the wild-type cell lines, the IC50 of MPA in Ish-MR cells increased significantly (Fig. S6B, C). Cellular functional experiments revealed that following overexpression of ROCK1, Ish-MR cells exhibited behaviors akin to Ishikawa cells, with significantly increased invasion and migration capacity (Fig. S7). Apoptosis experiments unveiled that ROCK1 overexpression resulted in diminished sensitivity of Ish-OR-ROCK and Ish-MR-OE-ROCK cells to progesterone-induced apoptosis, thereby implying reduced progesterone sensitivity (Fig. 7A, B). Interestingly, when Y-27632 was administered in conjunction with progesterone, the IC50 of MPA decreased, substantially elevating the apoptosis rate in Ish-MR cells compared to single-drug treatment (Fig. 7B). The clone formation experiment revealed that the combined application of Y-27632 and progesterone significantly inhibited cell clone formation, and the inhibition effect was higher than single-drug treatment (Figs. 7C and S8A). CCK-8 proliferation assay showed that cells with low ROCK1 expression were more sensitive to MPA (Fig. S7C, D). The results of cytoskeleton staining experiments showed that under the action of progesterone, the cytoskeleton of Ish-MR cells exhibited poor formation (Fig. 7D). In an in vivo context, Ishikawa cells were introduced into nude mice to form tumors. These mice were subsequently treated every alternate day with saline, progesterone, Y-27632, or a combination of progesterone and Y-27632. The combination therapy showed a more significant inhibitory effect on the tumors than the single-drug treatment (Figs. 7E and S8B). Immunohistochemical staining of subcutaneous tumors in nude mice showed that Ki-67 expression was highest in the saline group and lowest in the combination therapy group (Fig. 7F). However, compared with the control group, the body weight of mice in the progesterone treatment group increased significantly, while that in the Y-27632 treatment group decreased. The combined effect of the two drugs on the body weight of mice was not significant (Fig. 7G). These results suggest that Y-27632 enhances cell sensitivity to progesterone by inhibiting the activity of ROCK1.

## Discussion

ECM stiffness, as a critical modulator of cellular processes, exerts a profound influence on cell growth, differentiation, invasion, and migration capacities. Tumor ECM typically exhibits higher stiffness compared to healthy tissues (e.g., human healthy breast tissue approximately 400–1200 Pa, while invasive breast cancer tissue ranges from 5 to 200 kPa [46]), and this phenomenon is likely closely associated with tumor progression. In our study, we found that the stromal stiffness in EC patients was higher than that in normal endometrium. Building upon this observation, we conducted further research and identified ROCK1 as a gene strongly associated with ECM stiffness, with a robust correlation observed in EC. Importantly, we discovered that ROCK1 can drive EC progression by activating YAP1, and the application of its inhibitor, Y-27632, could mitigate EC advancement. Furthermore, of notable significance, we found that ROCK1 expression can serve as a predictive factor for treatment sensitivity in EC patients. Notably, our findings complement recent advances in EC mechanobiology. While Li et al. [47] demonstrated that traction force (TF) drives EC metastasis via SLC8A1/Wnt-β-catenin/F-actin signaling, our study reveals that ECM stiffness activates ROCK1/YAP1 to promote proliferation, therapy resistance, and immune evasion. Both works implicate cytoskeletal remodeling (F-actin) but diverge in mechanical triggers (TF vs. ECM stiffness) and functional outcomes (metastasis vs. treatment sensitivity) [47]. Critically, our work extends the therapeutic landscape by establishing ROCK1 as a target to reverse hormone/chemo-resistance and enhance macrophage phagocytosis—mechanisms previously unexplored in EC mechanobiology. These complementary insights position ROCK1/YAP1 inhibition as a strategy to augment existing anti-metastatic approaches. This study highlights the promising potential of ROCK1 as a prospective target for the diagnosis and treatment of EC.

The dysregulation of ECM within the tumor microenvironment is a prominent hallmark of cancer [48]. The mechanical properties conferred by the ECM, such as enhanced deposition or crosslinking, increase tissue matrix stiffness, which is closely associated with cancer proliferation, invasion, migration, and metastatic dissemination. Throughout cancer development, malignant cells contribute to ECM stiffening, while the rigid ECM, in turn, alters cancer cell characteristics. Notably, our investigation uncovered elevated ECM stiffening in EC patients compared to normal individuals, with a concomitant increase in cellulose components within EC tissue, substantiating the occurrence of EC stiffening. Prior research has indicated that ECM stiffening serves to upregulate ROCK1 expression. ROCK1, belonging to the AGC kinase family of Ser/Thr kinases, modulates cellular contractility via modulation of myosin phosphorylation [28–32]. Its role is context-dependent across diverse tumor types and stages, participating in diverse biological processes such as cellular fate determination and mechanotransduction, and correlating with malignant behaviors [32, 49–51]. In the context of EC, ROCK1 emerges as a pivotal mechanosensitive gene, attaining higher expression in EC patients and correlating with adverse prognosis. Immunophenoscore analysis has unveiled that heightened ROCK1 expression correlates with increased tumor cellularity and diminished tumor-infiltrating immune cells, further underscoring the potential of ROCK1 as an adverse prognostic factor. Notably, modulation of cell culture substrate stiffness demonstrated that ROCK1 expression escalates in tandem with substrate stiffness augmentation within EC. Furthermore, cellular experiments show that ROCK1 expression increased with enhanced matrix stiffness. Additionally, cells with elevated ROCK1 expression demonstrated accelerated proliferation, facilitated G2-phase cell cycle progression, reduced apoptosis, and increased invasion and migration capabilities. Consequently, we postulate that ECM stiffening may precipitate heightened ROCK1 expression, thereby fostering the progression of EC.

YAP1, a crucial regulatory factor for cell growth and differentiation activated by matrix stiffening, emerged from our investigation into the mechanism of ROCK1-induced EC progression. Within this context, an interaction between ROCK1 and YAP1 was revealed. YAP1, an integral member of the Src family kinases, assumes a pivotal role as a transcriptional co-activator within the Hippo signaling pathway [52]. Its primary association with the TEAD transcription factor family sets in motion a cascade of gene expression programs critical for a multitude of biological processes. YAP1 exhibits indispensable functions in cell proliferation, tissue regeneration, mechanotransduction, tissue and organ homeostasis, as well as cancer metastasis. Notably, non-phosphorylated YAP1 undergoes translocation to the nucleus, where it engages with TEAD transcription factors, while phosphorylation events render YAP1 inactive, hindering its nuclear translocation [53]. Our investigations revealed that increased ROCK1 expression affects the phosphorylation of YAP1, thereby influencing its activity. Moreover, we found that knocking down YAP1 partially rescues the biological effects induced by ROCK1 overexpression. Prior research had indicated YAP1 activation under conditions of cultivation on rigid substrates [54]. Our study, in contrast, highlights that alterations in substrate stiffness in EC cells did not significantly impact YAP1 expression levels. Therefore, we posit that alterations in matrix stiffness in EC do not directly impact the expression of YAP1 but instead exert the biological effects by modulating the phosphorylation status of YAP1 through the expression of ROCK1.

Tumor metastasis necessitates the reconstruction of the cellular cytoskeleton, a process profoundly influenced by the mechanical forces arising from ECM stiffening. Cytoskeletal rearrangement is essential for cell movement, and actin polymerization and depolymerization occur in concert [55]. There are numerous signaling pathways involved in this process, with the Rho/ROCK pathway being one of the most well-known [56]. In our investigation, we found that EC cell lines with elevated ROCK1 expression exhibited elongated and well-organized actin filaments. Prior research has indicated that when mechanical forces are exerted on cancer cells, actin filaments function as mechanosensors, inducing intracellular signal transduction pathways. Actin II interacts to generate contractile forces, propelling the cytoplasmic membrane forward through polymerization [57]. Our study discovered that despite unaffected ROCK1 expression, inhibition of its function via the ROCK1 inhibitor Y-27632 resulted in notable outcomes. Proliferation and migration of uterine EC cell lines were significantly suppressed. Concomitantly, actin filaments within the cells markedly decreased in a concentration- and time-dependent manner. We postulate that ECM stiffening, by modulating ROCK1, induces actin filament formation, consequently promoting the migration of EC cells and thereby driving disease progression. Intriguingly, Y-27632 effectively inhibited the malignant phenotype, fostering cytoskeletal reorganization. These findings propose Y-27632 as a potential therapeutic agent for uterine EC treatment.

Furthermore, ECM stiffening affects drug penetration [58], genomic stability [59, 60], and immune cell infiltration [61, 62], thereby impacting tumor treatment efficacy. ROCK1 has been shown to regulate immune cell infiltration and treatment sensitivity [31, 63]. Thus, we explored the implications of ROCK1 expression in guiding EC treatment decisions. Traditionally, surgery is the primary treatment for EC, followed by adjuvant therapy based on staging and high-risk factors, including radiation therapy, chemotherapy, immunotherapy, and hormone therapy. In this study, we mainly focused on conventional adjuvant treatment strategies, including radiotherapy, chemotherapy, and hormone therapy. Our research revealed that EC patients with low ROCK1 expression may demonstrate heightened sensitivity to radiation therapy, while those with high ROCK1 expression may be more responsive to chemotherapy. Both radiation and chemotherapy can induce immunogenic cell death (ICD) [64, 65], promoting a robust antitumor immune response and enhancing treatment outcomes [65, 66]. Surprisingly, in the presence of the ROCK1 inhibitor Y-27632, macrophages exhibited augmented phagocytic activity against tumor cells following radiation-induced ICD. Therefore, we believe that Y-27632 can enhance the immune therapeutic response. Additionally, building on our prior work demonstrating YAP1 [67] as a key mediator of hormone therapy response, we investigated the impact of ROCK1 expression on hormone therapy sensitivity. We found that cells with high ROCK1 expression displayed reduced sensitivity to low-dose progesterone therapy, and Y-27632 restored progesterone sensitivity by suppressing ROCK1-driven YAP1 activation. This synergy highlights ROCK1/YAP1 as a critical axis bridging mechanical signaling and hormonal resistance. Notably, Xuerun et al. recently identified estrogen-driven EC progression via calcium influx-mediated cytoskeletal remodeling, proposing calcium channel blockers as adjuvants [68]. While their work focuses on estrogen-calcium crosstalk, our study complements this by defining ROCK1/YAP1 as a progesterone-resistance hub under stiff ECM conditions. These parallel insights advocate dual targeting of hormonal (e.g., progesterone/AIs) and mechanical (ROCK inhibitors) pathways to overcome EC therapy resistance.

Mounting preclinical evidence supports ROCK inhibition as a versatile strategy to augment diverse cancer therapies. In pancreatic adenocarcinoma, fasudil enhances gemcitabine uptake and extends survival when combined with chemotherapy [69]. Similarly, Y-27632 synergizes with doxorubicin in colorectal and melanoma models by amplifying antitumor immunity [70], while disrupting bone marrow niches to sensitize multiple myeloma to bortezomib [71]. ROCK inhibitors also overcome targeted therapy resistance: Y-27632, GSK269962A, or fasudil restore BRAF inhibitor efficacy in melanoma, and ROCK1 silencing sensitizes BRAF/NRAS-mutant cells to MAPK inhibitors [72–74]. The ROCK1/2 inhibitor RKI-1447 suppresses N-MYC, induces cell death, and synergizes with BET inhibitors in neuroblastoma [75]. These cross-cancer applications underscore the translational promise of ROCK-targeted combinatorial approaches, aligning with our EC findings. While over 170 ROCK inhibitors are under development or clinically approved (e.g., fasudil, ripasudil), only one has entered oncology trials, limited by narrow therapeutic indices, poor tumor penetration, and adverse reactions (e.g., hypotension, headaches) [76, 77]. Our study employed Y-27632, while emerging next-generation inhibitors (e.g., indazole/pyridine-derived compounds) exhibit enhanced ROCK specificity and reduced AGC kinase family cross-reactivity [76, 78], offering promise for EC applications. Future efforts should prioritize optimizing pharmacokinetics and tumor targeting while minimizing vascular toxicity. Clinical validation of selective ROCK1 inhibitors, particularly in combination with hormonal or immunotherapies, could unlock their full potential in EC management, building upon our mechanistic insights into the ROCK1/YAP1 axis. Clinical validation of selective ROCK1 inhibitors, particularly in combination with hormonal or immunotherapies, may unlock their potential in EC management, building upon our mechanistic insights into the ROCK1/YAP1 axis. In preclinical models, we observed that Y-27632 monotherapy modestly suppressed weight gain in mice, while co-administration with progesterone attenuated progesterone-induced weight gain—a side effect commonly associated with hormonal therapies. These preliminary findings suggest that ROCK1 inhibition could offer dual advantages in enhancing therapeutic efficacy and mitigating metabolic adverse effects, though further studies are needed to validate these observations in clinical settings. Future trials exploring ROCK1 inhibitors in combination with hormonal agents should prioritize both efficacy and tolerability to refine EC treatment strategies.

In summary, our research reveals that EC patients exhibit ECM stiffening, which is associated with increased ROCK1 expression. The ROCK1/YAP1 signaling pathway plays a crucial role in influencing the aggressive behavior of EC. Additionally, ROCK1 expression can guide treatment decisions for EC patients. Those with low ROCK1 expression may benefit from radiation therapy, while those with high ROCK1 expression may respond better to chemotherapy. Furthermore, the use of the ROCK1 inhibitor Y-27632 enhances the phagocytic activity of macrophages and improves the sensitivity of tumor cells to hormone therapy. Therefore, targeted therapies targeting ROCK1 hold promise for improving the treatment outcomes of EC patients.

## Supplementary information

Supplementary figures and original WB images

## Data Availability

The open-source dataset analyzed in this study was downloaded from the TCGA repository. Other data generated and analyzed in the study are included in this published article. Additional data and materials of the study can be obtained from the corresponding author.
