# Supplementary material for: Extracellular matrix stiffness in endometrial cancer: driving progression and modulating treatment sensitivity via the ROCK1/YAP1 axis
Source: Cell Death Dis. 2025 May 14;16(1):380. doi: 10.1038/s41419-025-07697-8 (PMC12078694; doi:10.1038/s41419-025-07697-8)
Supplement: Supplementary file 1 — Supplementary figures and original WB images [file 41419_2025_7697_MOESM1_ESM.pdf]

Supplementary figures

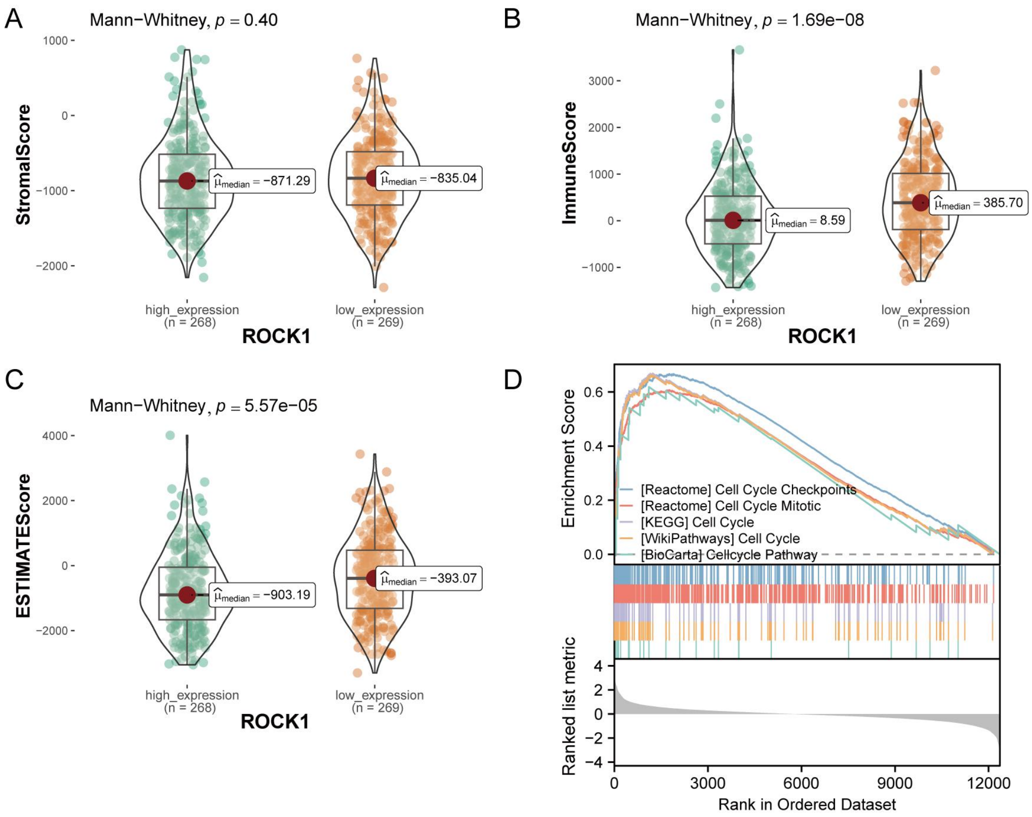

Figure S1 Estimate and GSEA analysis of EC patients according to ROCK1 expression.

A . Estimate algorithm revealed no significant difference in the stromal score between patients with high ROCK1 expression and those with low ROCK1 expression.

B . Patients with low ROCK1 expression exhibited higher immune scores.

C . Patients with low ROCK1 expression had higher Estimate score.

D . Patients with high ROCK1 expression were enriched in cell cycle related pathways.

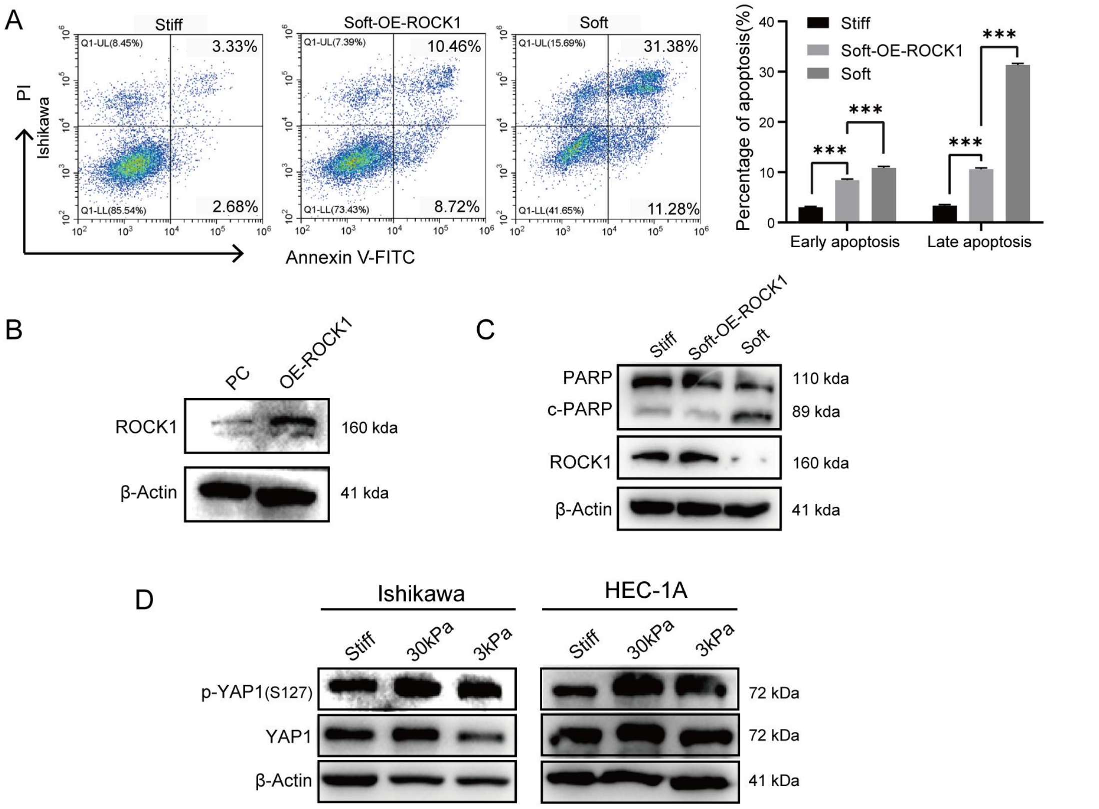

Figure S2 Cell apoptosis and protein expression cells cultured under different stiffness conditions.

A. Cell apoptosis of Ishikawa with different ROCK1 expression cultured on stiff and soft substrate.

B. Western blot analysis to detect protein efficiency of ROCK1 overexpression in Ishikawa cells.

C. Related protein expression of Ishikawa with different ROCK1 expression cultured on stiff and soft substrate.

D. YAP1 expression of Ishikawa and HEC-1A cells cultured with different stiffness substrate.

Note: The β-actin band for the HEC-1A group in this panel is reused from Figure 2D, as both panels represent the same sample under identical stiffness conditions (Stiff, 30 kPa, and 3 kPa).

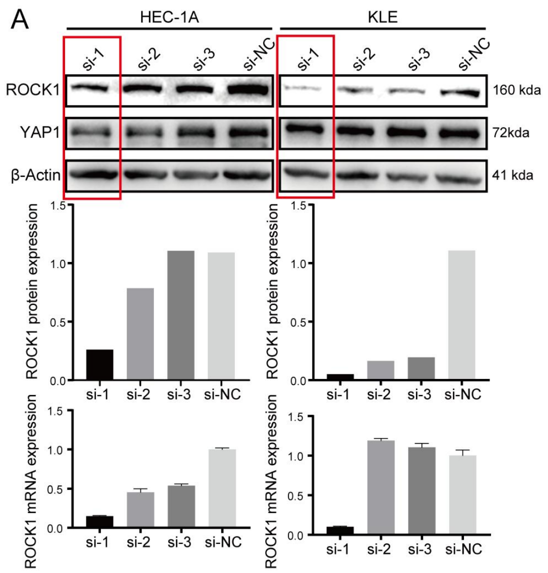

Figure S3 Efficiency of ROCK1 knockdown and overexpression.

A． Comparison of the knockdown efficiency of 3 ROCK1-targeting siRNA on ROCK1 protein and mRNA expression in HEC-1A and KLE cells (the most efficient siRNA marked in red).

B． ROCK1 mRNA expression after ROCK1 knockdown in HEC-1A cells and ROCK1 overexpression in Ishikawa, Ish-MR, and HEC-1A cells. **: *P*<0.01, ***: *P*<0.001.

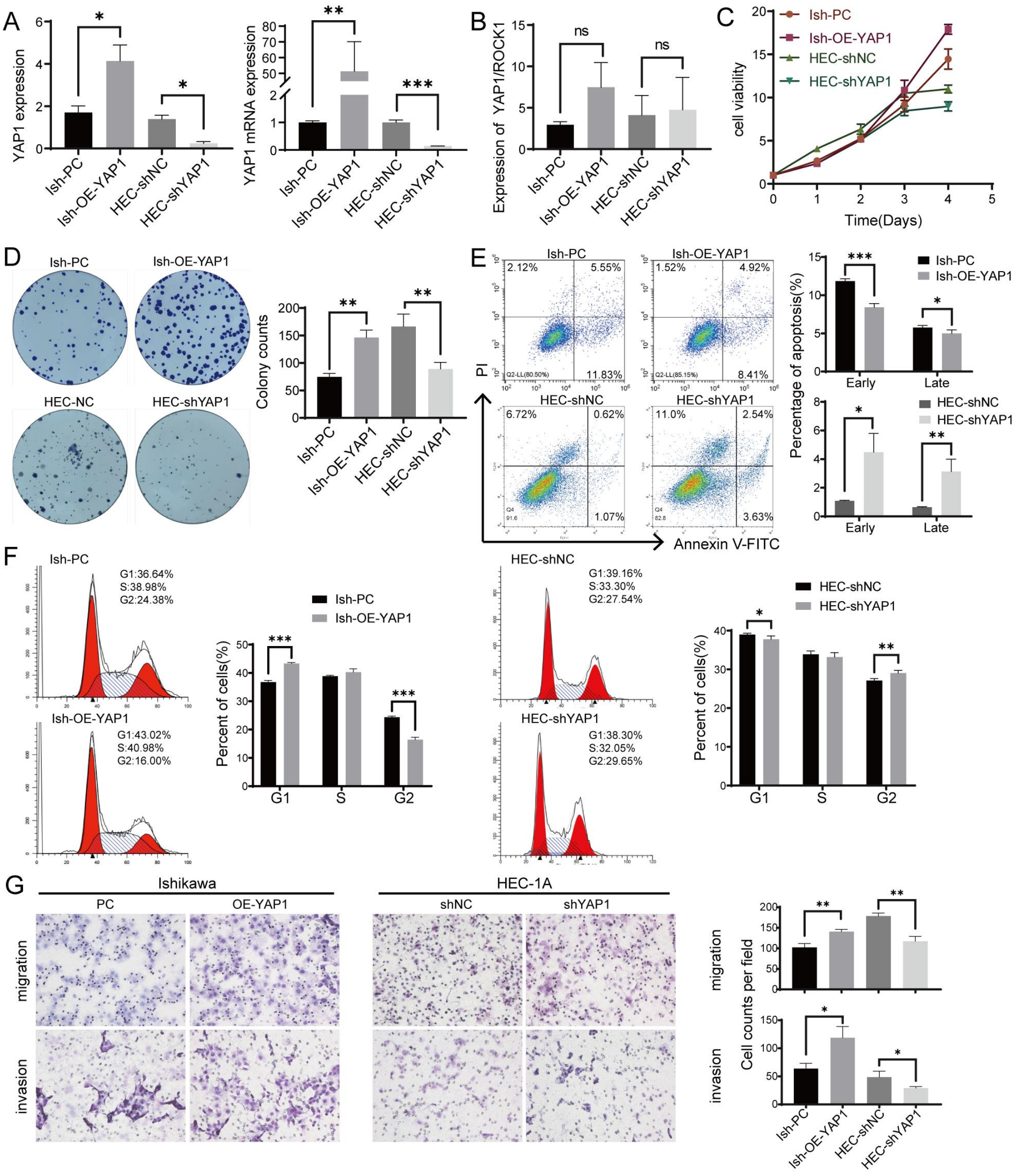

Figure S4 Elevated YAP1 expression contributes to the malignant biological behavior of endometrial cancer cells.

A . Protein and mRNA expression profiles of YAP1 in YAP1-overexpressed and knocked-down cells (statistical graphs).

B . Expression of YAP1/ROCK1 profiles after overexpression and knockdown of YAP1.

C . Proliferation assay of Ishikawa and HEC-1A cells after YAP1 overexpression and knockdown.

D . Clonogenic assay depicting the colony formation of cells in YAP1 overexpressed and knock-down conditions.

E . Assessment of apoptosis ratio in cells with YAP1 overexpression and knockdown.

F . Cell cycle distribution analysis in cells with YAP1 overexpression and knockdown.

G . Transwell assay to evaluate the invasion and migration capabilities of cells after YAP1 overexpression and knockdown. *: *P*<0.05, **: *P*<0.01, ***: *P*<0.001.

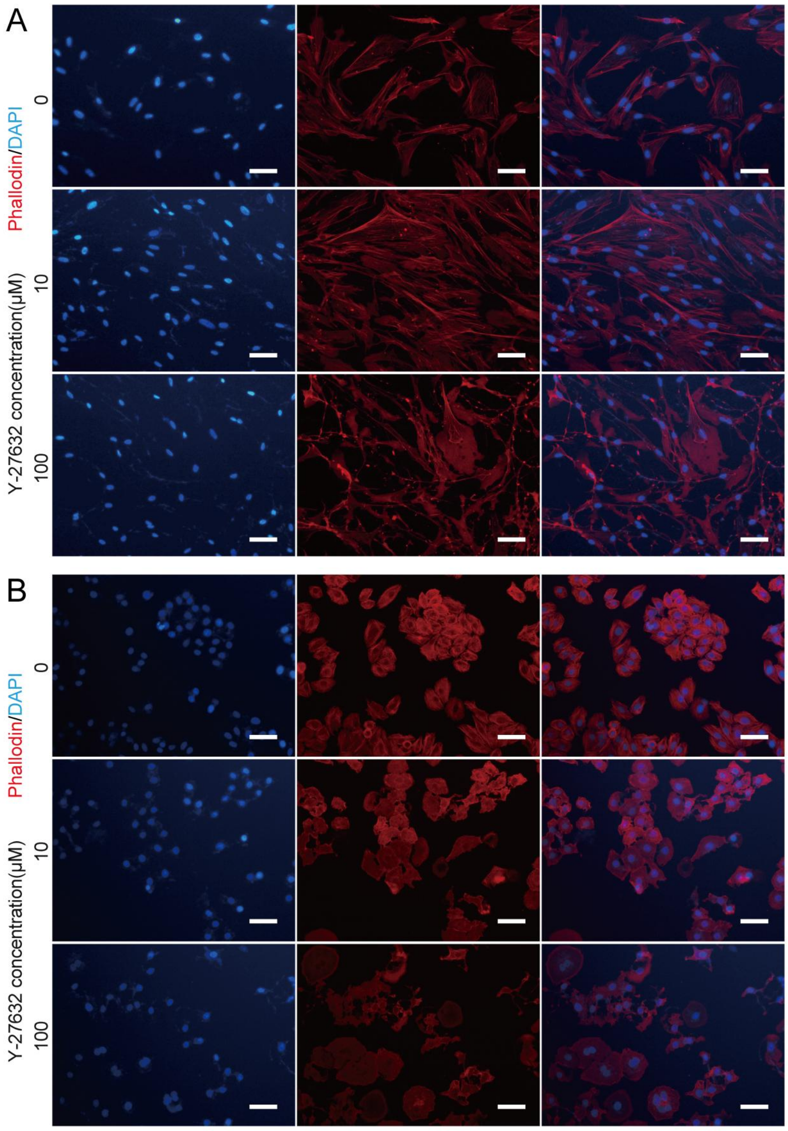

Figure S5 Cytoskeleton staining of hESCs and Ish-MR cells with Y-27632 treatment.

A . Immunofluorescence staining of the cytoskeleton in hESCs cells (human endometrial stromal cell line) after treatment with 0, 10, and 100 μM Y-27632.

B . Immunofluorescence staining of the cytoskeleton in Ish-MR cells after treatment with 0, 10, and 100 μM Y-27632. Scale: 50 micrometers.

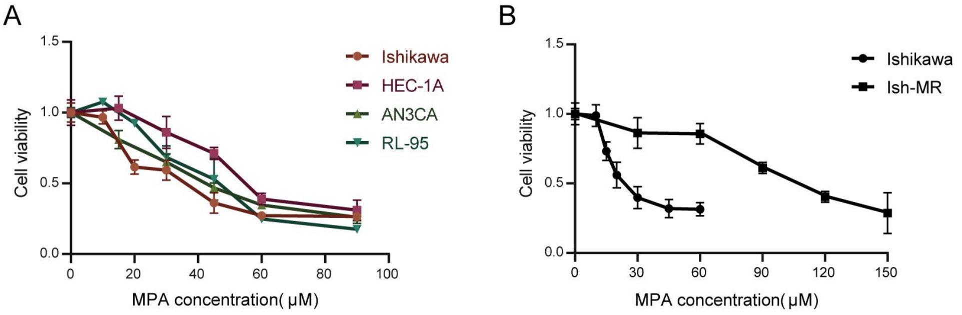

C

Cell	Ishikawa	HEC-1A	AN3CA	RL-95	Ish-MR
IC50（μM）	24.03	46.59	39.06	38.70	99.09

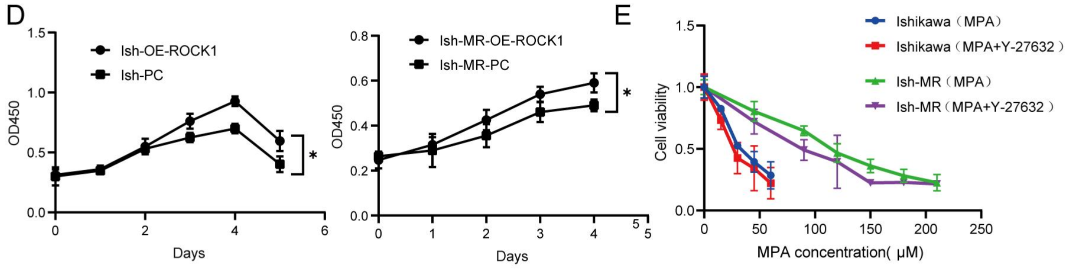

Figure S6 Cell proliferation assay to detect the effect of MPA.

A． IC50 of MPA in 4 endometrial cancer cell lines.

B． IC50 of MPA in Ishikawa and Ish-MR cells.

C． The IC50 value of cell lines.

D． The effect of ROCK1 overexpression on cell proliferation was compared under the treatment of MPA.

E． IC50 of Ishikawa and Ish-MR cells with different ROCK1 expression after treated with combination of Y-27632 and MPA. *: P<0.05.

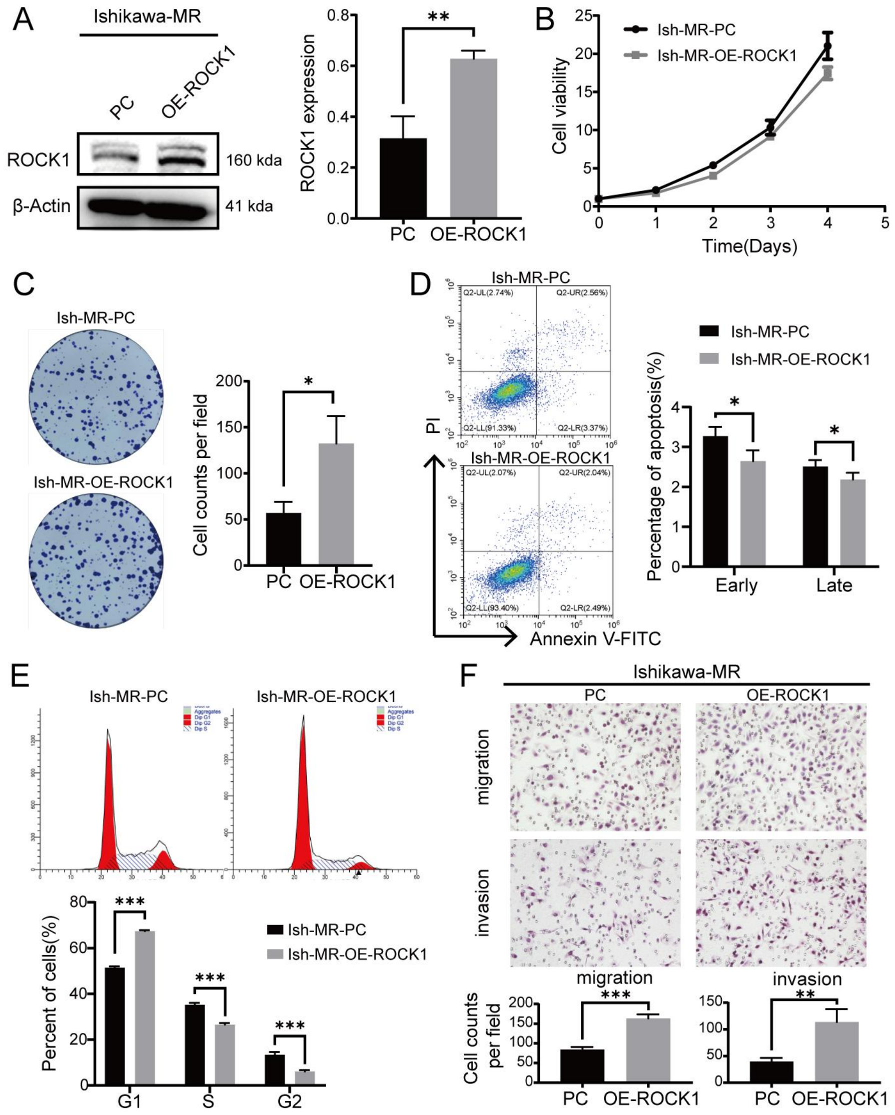

Figure S7 Elevated ROCK1 expression contributes to the malignant biological behavior of progesterone resistance endometrial cancer cells.

F． Western blot analysis to detect protein efficiency of ROCK1 overexpression in Ish-MR cells.

G． Proliferation of Ish-MR cells after ROCK1 overexpression.

H． Clonogenic formation of Ish-MR cells after ROCK1 overexpression.

I． Apoptosis of Ish-MR cells after ROCK1 overexpression.

J． Cell cycle distribution of Ish-MR cells after ROCK1 overexpression.

K． Migration and invasion of Ish-MR cells after ROCK1 overexpression assessed by Transwell assay. *: *P*<0.05, **: *P*<0.01, ***: *P*<0.001.

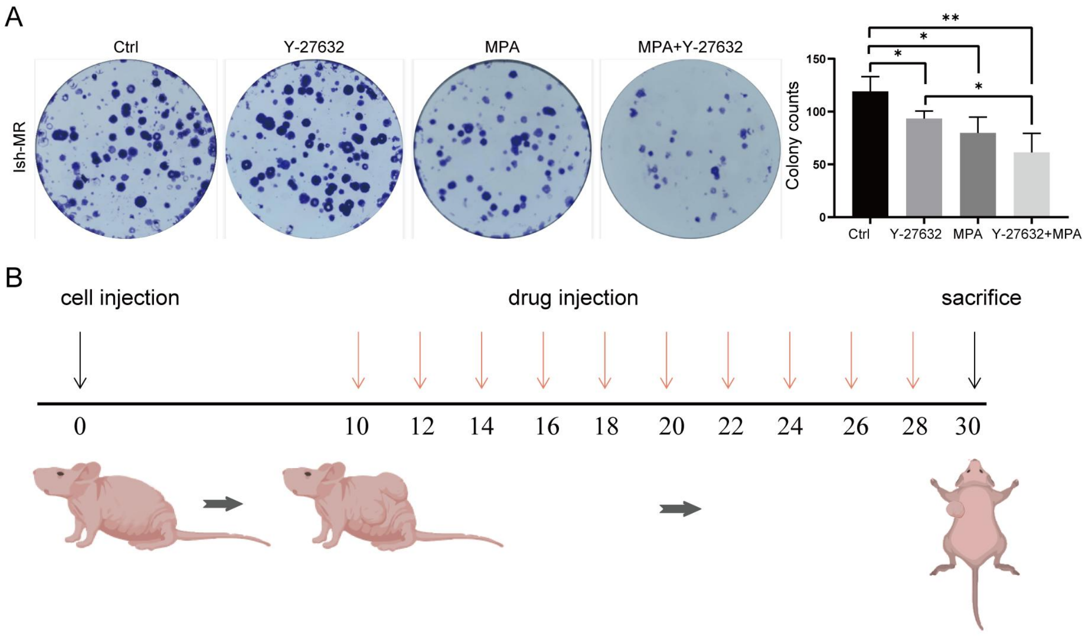

Figure S8 Colony formation assay and in vivo xenograft experiment of endometrial cancer cells.

A． Colony formation assay of Ish-MR cells treated with MPA, Y-27632, or their combination.

B． Schematic diagram of in vivo xenograft formation and drug administration in nude mice.

Original original Western blot images

Fig 2

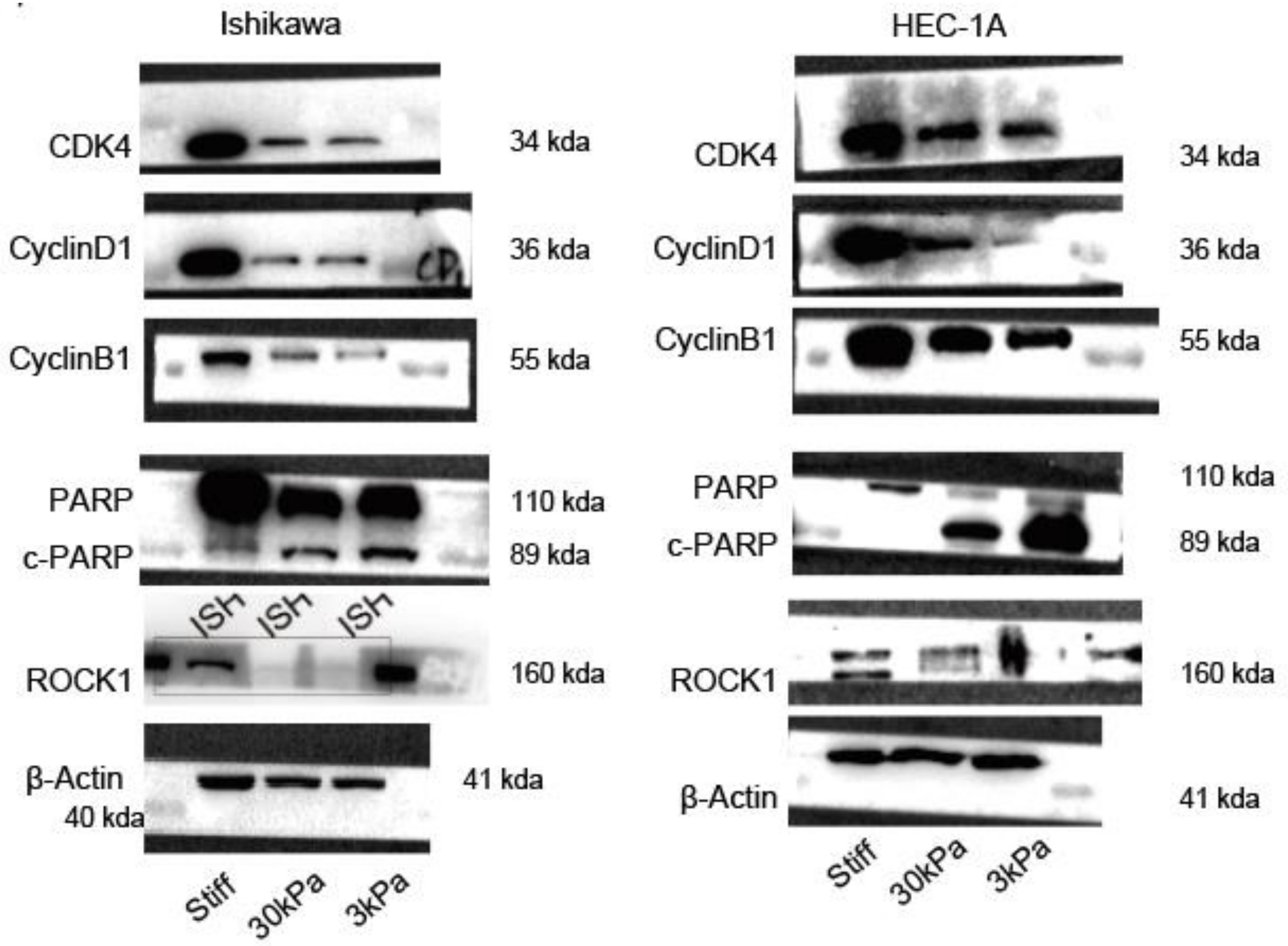

Fig 3

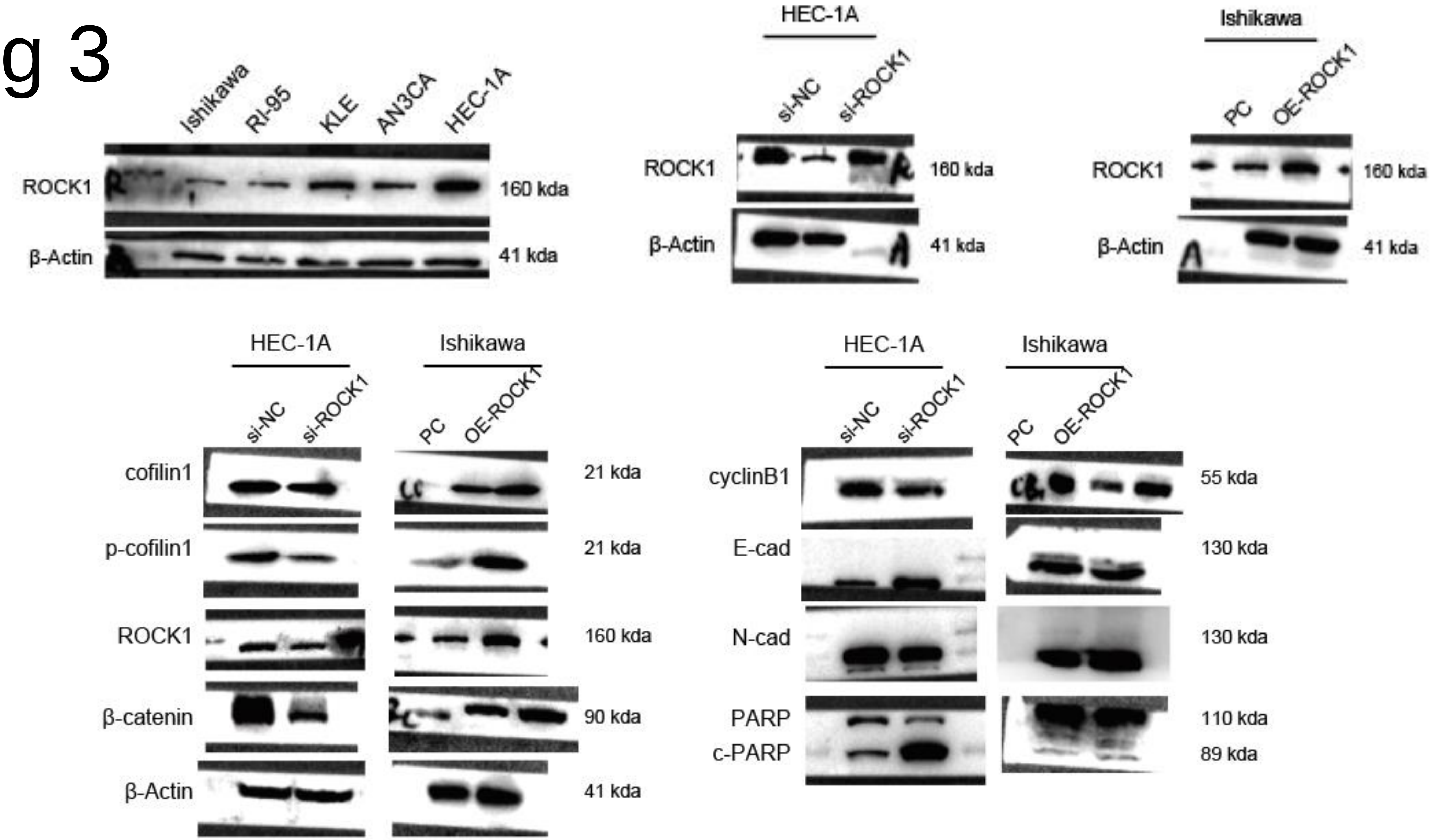

Ishikawa
RI-95
KLE
AN3CA
HEC-1A
ROCK1
160 kda
β-Actin
41 kda
HEC-1A
si-NC
si-ROCK1
ROCK1
160 kda
β-Actin
41 kda
Ishikawa
PC
OE-ROCK1
ROCK1
160 kda
β-Actin
41 kda
HEC-1A
Ishikawa
si-NC
si-ROCK1
PC
OE-ROCK1
cofilin1
21 kda
p-cofilin1
21 kda
ROCK1
160 kda
β-catenin
90 kda
β-Actin
41 kda
HEC-1A
Ishikawa
si-NC
si-ROCK1
PC
OE-ROCK1
cyclinB1
55 kda
E-cad
130 kda
N-cad
130 kda
PARP
110 kda
c-PARP
89 kda

Fig 4

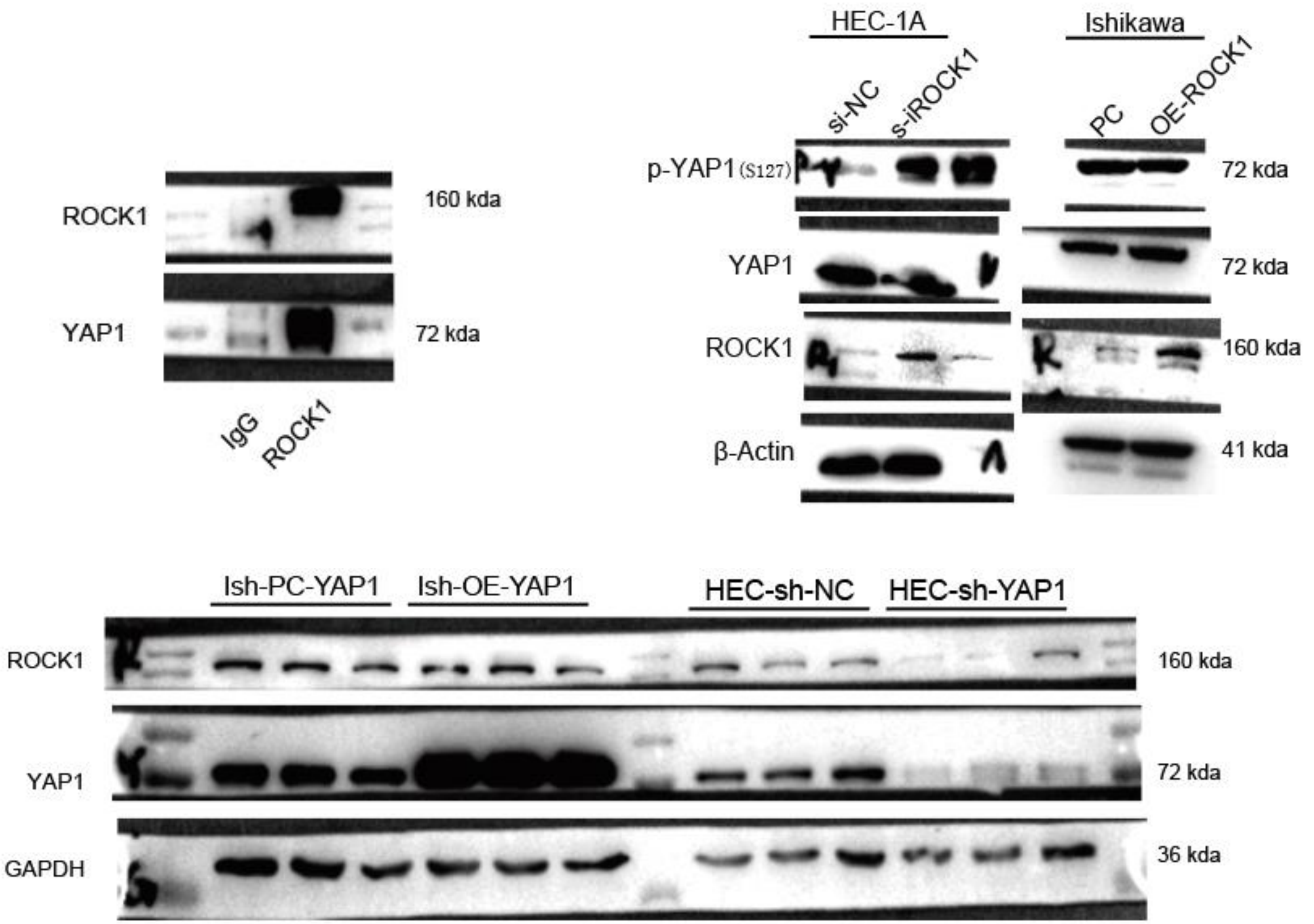
ROCK1
YAP1
160 kda
72 kda
IgG
ROCK1
HEC-1A
Ishikawa
si-NC
s-iROCK1
PC
OE-ROCK1
p-YAP1 (S127)
YAP1
ROCK1
β-Actin
72 kda
72 kda
160 kda
41 kda
Ish-PC-YAP1
Ish-OE-YAP1
HEC-sh-NC
HEC-sh-YAP1
ROCK1
YAP1
GAPDH
160 kda
72 kda
36 kda

Fig 5

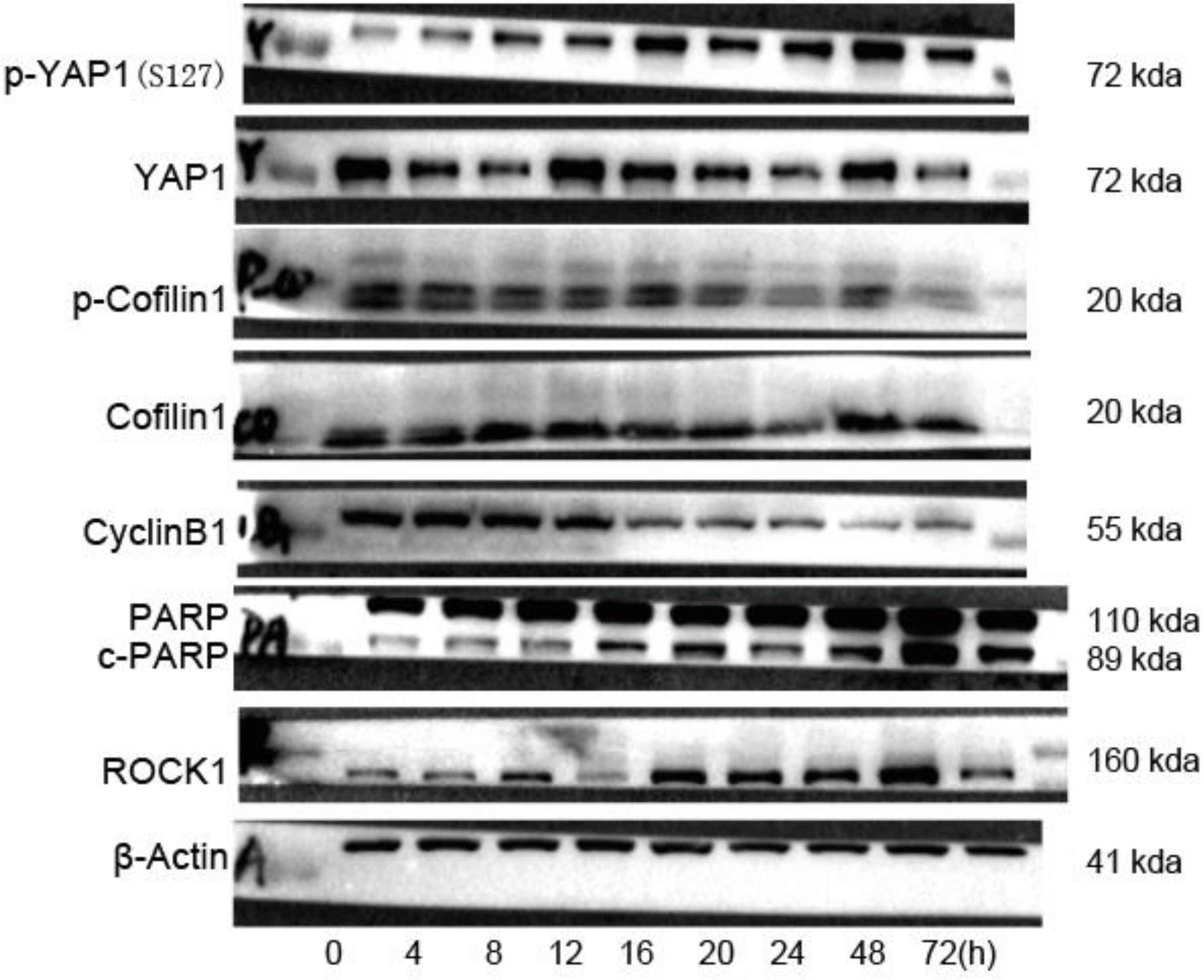
p-YAP1 (S127)
72 kda
YAP1
72 kda
p-Cofilin1
20 kda
Cofilin1
20 kda
CyclinB1
55 kda
PARP
c-PARP
110 kda
89 kda
ROCK1
160 kda
β-Actin
41 kda
0 4 8 12 16 20 24 48 72(h)

Fig 6

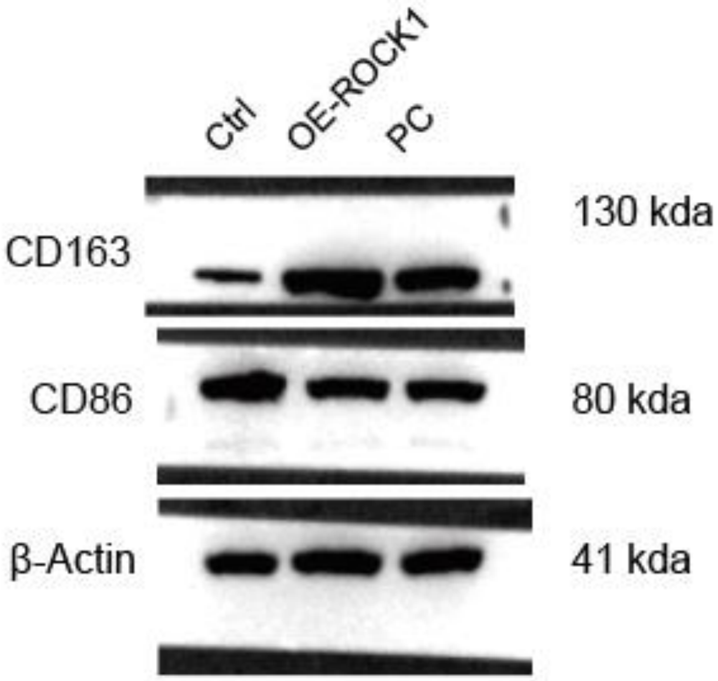

Fig S2

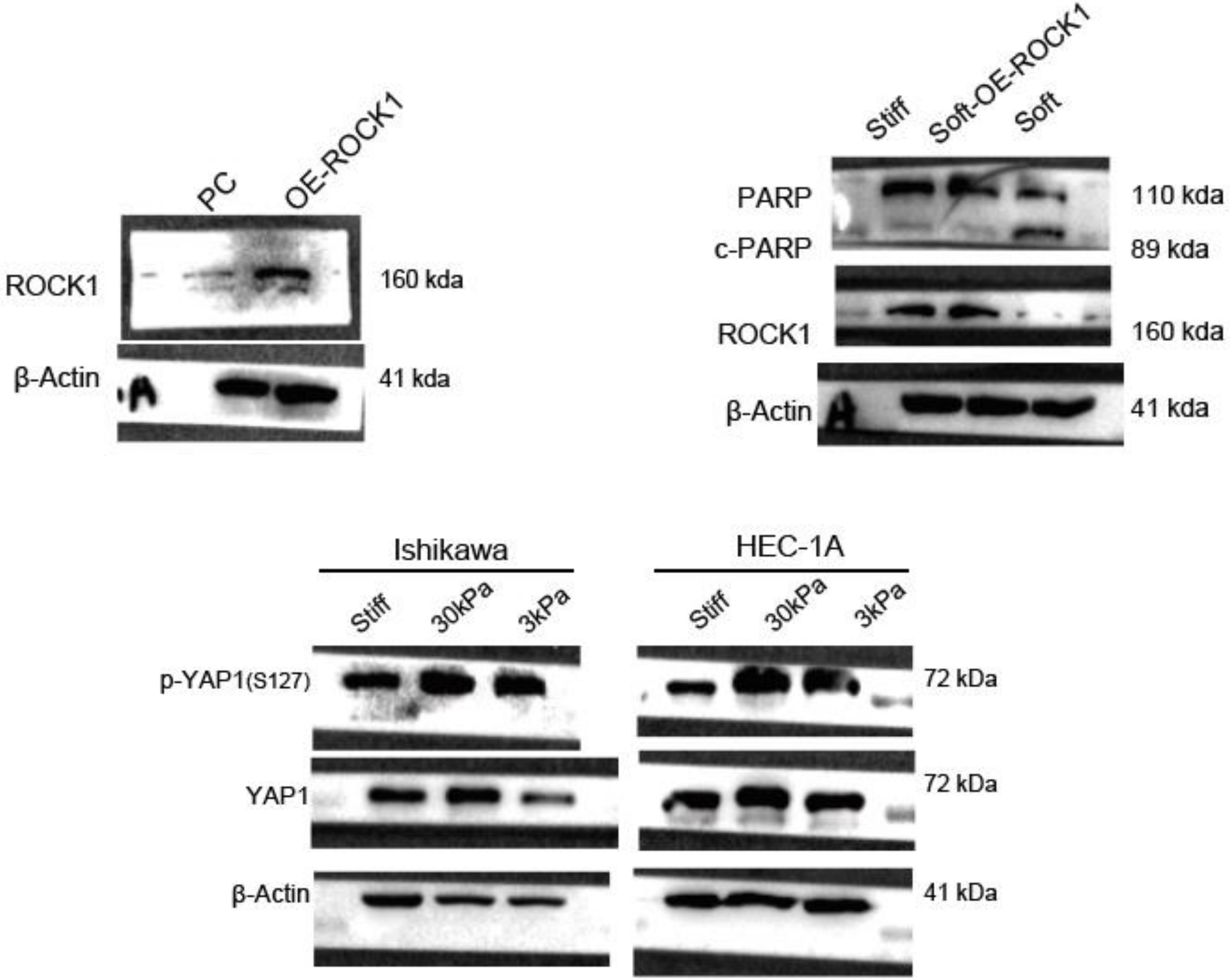
PC
OE-ROCK1
ROCK1
160 kda
β-Actin
41 kda
Stiff
Soft-OE-ROCK1
Soft
PARP
c-PARP
110 kda
89 kda
ROCK1
160 kda
β-Actin
41 kda
Ishikawa
HEC-1A
Stiff
30kPa
3kPa
Stiff
30kPa
3kPa
p-YAP1(S127)
72 kDa
YAP1
72 kDa
β-Actin
41 kDa

Fig S3

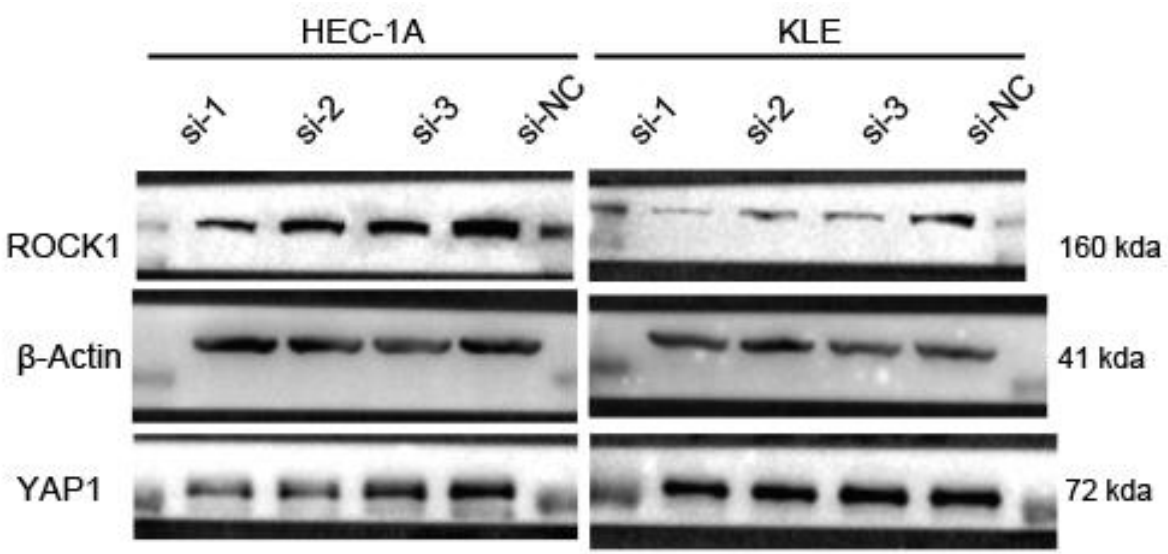

HEC-1A
KLE
si-1
si-2
si-3
si-NC
si-1
si-2
si-3
si-NC
ROCK1
β-Actin
YAP1
160 kda
41 kda
72 kda

Fig S7

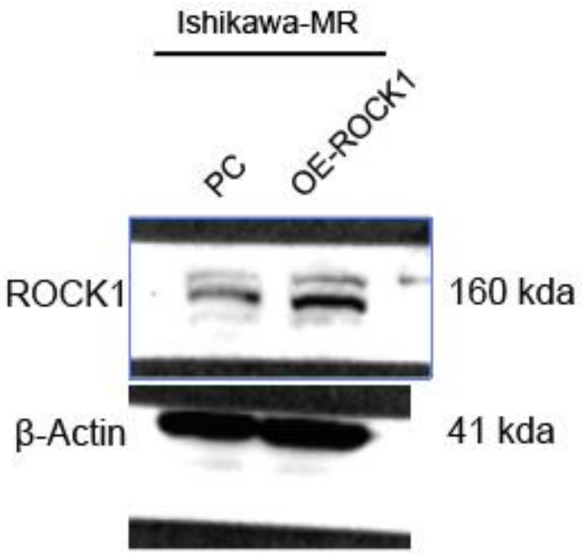

Ishikawa-MR
PC
OE-ROCK1
ROCK1
β-Actin
160 kda
41 kda
